# Resist or perish: Fate of a microbial population subjected to a periodic presence of antimicrobial

**DOI:** 10.1371/journal.pcbi.1007798

**Published:** 2020-04-10

**Authors:** Loïc Marrec, Anne-Florence Bitbol

**Affiliations:** 1 Sorbonne Université, CNRS, Institut de Biologie Paris-Seine, Laboratoire Jean Perrin (UMR 8237), F-75005 Paris, France; 2 Institute of Bioengineering, School of Life Sciences, École Polytechnique Fédérale de Lausanne (EPFL), Lausanne, Switzerland; Rutgers University, UNITED STATES

## Abstract

The evolution of antimicrobial resistance can be strongly affected by variations of antimicrobial concentration. Here, we study the impact of periodic alternations of absence and presence of antimicrobial on resistance evolution in a microbial population, using a stochastic model that includes variations of both population composition and size, and fully incorporates stochastic population extinctions. We show that fast alternations of presence and absence of antimicrobial are inefficient to eradicate the microbial population and strongly favor the establishment of resistance, unless the antimicrobial increases enough the death rate. We further demonstrate that if the period of alternations is longer than a threshold value, the microbial population goes extinct upon the first addition of antimicrobial, if it is not rescued by resistance. We express the probability that the population is eradicated upon the first addition of antimicrobial, assuming rare mutations. Rescue by resistance can happen either if resistant mutants preexist, or if they appear after antimicrobial is added to the environment. Importantly, the latter case is fully prevented by perfect biostatic antimicrobials that completely stop division of sensitive microorganisms. By contrast, we show that the parameter regime where treatment is efficient is larger for biocidal drugs than for biostatic drugs. This sheds light on the respective merits of different antimicrobial modes of action.

## Author summary

Antimicrobials select for resistance, which threatens to make antimicrobials useless. Understanding the evolution of antimicrobial resistance is therefore of crucial importance. Under what circumstances are microbial populations eradicated by antimicrobials? Conversely, when are they rescued by resistance? We address these questions employing a stochastic model that incorporates variations of both population composition and size. We consider periodic alternations of absence and presence of antimicrobial, which may model a treatment. We find a threshold period above which the first phase with antimicrobial fully determines the fate of the population. Faster alternations strongly select for resistance, and are inefficient to eradicate the microbial population, unless the death rate induced by the treatment is large enough. For longer alternation periods, we calculate the probability that the microbial population gets eradicated. We further demonstrate the different merits of biostatic antimicrobials, which prevent sensitive microbes from dividing, and of biocidal ones, which kill sensitive microbes.

## Introduction

Antibiotics and antivirals allow many major infectious diseases to be treated. However, with the increasing use of antimicrobials, pathogenic microorganisms tend to become resistant to these drugs, which then become useless. Understanding the evolution of resistance is of paramount importance in order to fight the major public health issue raised by antimicrobial resistance [1, 2].

The evolution of antimicrobial resistance often occurs in a variable environment, as antimicrobial is added and removed from a medium or given periodically to a patient [3, 4]. This results into varying patterns of selection, which are known to have a dramatic effect on evolution in other contexts [5–9]. To address how variations of antimicrobial concentration impact resistance evolution, we investigate theoretically the *de novo* acquisition of resistance in a microbial population in the presence of alternations of phases of presence and absence of antimicrobial. This situation can represent, for example, a treatment where the concentration within the patient falls under the Minimum Inhibitory Concentration (MIC) between drug intakes [10], which is a realistic case [10, 11].

We propose a general stochastic model that incorporates variations of both population composition and size, i.e. population genetics and population dynamics. Despite having a common origin in stochastic birth, death and mutation events, and thus being intrinsically coupled, these phenomena are seldom considered together in theoretical studies [12]. However, it is particularly crucial to address both of them when studying the evolution of antimicrobial resistance, because the aim of an antimicrobial treatment is to eradicate a microbial population, or at least to substantially decrease its size, while the evolution of resistance corresponds to a change in the genetic makeup of the population. Our general model allows us to fully incorporate the stochasticity of mutation occurrence and establishment [13–17], as well as that of population extinction, whose practical importance was recently highlighted [18–20].

In this framework, we ask whether a microbial population subject to alternations of phases of presence and absence of antimicrobial develops resistance, which corresponds to treatment failure and to rescue of the microbial population by resistance [21, 22], or goes extinct, which corresponds to treatment success. In other words, we ask whether the microbial population resists or perishes.

We study both the impact of biocidal drugs, that kill microorganisms, and of biostatic drugs, that prevent microorganisms from growing. We show that fast alternations of phases with and without antimicrobial do not permit eradication of the microbial population before resistant mutants fix, unless the death rate with antimicrobial is large enough. Conversely, intermediate alternation speeds are effective for a wider range of antimicrobial modes of action, but the probability of population extinction and therefore of treatment success, which we fully quantify, is not one, because resistance can rescue the population, and this effect depends on the size of the microbial population. We find that the parameter range where antimicrobial treatment is efficient is larger for biocidal drugs than for biostatic drugs. However, we also show that biocidal and imperfect biostatic antimicrobials permit an additional mechanism of rescue by resistance compared to biostatic drugs that completely stop growth. This sheds light on the respective merits of different antimicrobial modes of action. Finally, we find a population size-dependent critical drug concentration below which antimicrobials cannot eradicate microbial populations.

## Model and methods

We consider a microbial population with carrying capacity *K*, corresponding to the maximum population size that the environment can sustain, given the nutrients available. The division rate of each microorganism is assumed to be logistic, and reads *f*(1 − *N*/*K*), where *N* represents the total population size, while the fitness *f* is the maximal division rate of the microorganism, reached when *N* ≪ *K*. This model therefore incorporates population size variations, and allows us to include extinctions induced by the antimicrobial drug.

Mutations that confer antimicrobial resistance are often associated with a fitness cost, i.e. a slower reproduction [23–25], but this fitness cost can be compensated by subsequent mutations [26–29]. The acquisition of resistance is therefore often irreversible, even if the antimicrobial is removed from the environment [24, 26]. Thus motivated, we consider three types of microorganisms: sensitive (S) microorganisms, whose division or death rate is affected by antimicrobials, resistant (R) microorganisms, that are not affected by antimicrobials but that bear a fitness cost, and resistant-compensated (C) microorganisms that are not affected by antimicrobials and do not bear a fitness cost. In the absence of antimicrobial, their fitnesses (maximal division rates) are denoted by *f*_*S*_, *f*_*R*_ and *f*_*C*_, respectively, and their death rates by *g*_*S*_, *g*_*R*_ and *g*_*C*_. Values in the presence of antimicrobial are denoted by a prime, e.g. $f_{S}^{\prime }$. Note that we include small but nonzero baseline death rates, which can model losses or the impact of the immune system *in vivo*, and allows for population evolution even at steady-state size. Without loss of generality, we set *f*_*S*_ = 1 throughout. In other words, the maximum reproduction rate of S microorganisms, attained when population size is much smaller than the carrying capacity, sets our time unit. We further denote by *μ*_1_ and *μ*_2_ the mutation probabilities upon each division for the mutation from S to R and from R to C, respectively. In several actual cases, the effective mutation rate towards compensation is higher than the one towards the return to sensitivity, because multiple mutations can compensate for the initial cost of resistance [27, 28, 30]. Thus, we do not take into account back-mutations. Still because of the abundance of possible compensatory mutations, often *μ*_1_ ≪ *μ*_2_[27, 31]. We provide general analytical results as a function of *μ*_1_ and *μ*_2_, and we focus more on the limit *μ*_1_ ≪ *μ*_2_, especially in simulations.

Our model thus incorporates both population dynamics and population genetics [7, 12, 32], and is more realistic than descriptions assuming constant population sizes [33], e.g. in the framework of the Moran process [13, 34]. Throughout, our time unit corresponds to a generation of sensitive microorganisms without antimicrobial in the exponential phase (reached when *N* ≪ *K*).

The action of an antimicrobial drug can be quantified by its MIC, which corresponds the minimum concentration that stops the growth of a microbial population [24]. More precisely, the MIC corresponds to the concentration such that death rate and division rate are equal [18]: in a deterministic framework, above the MIC, the population goes extinct, while below it, it grows until reaching carrying capacity. We investigate the impact of periodic alternations of phases of absence and presence of antimicrobial, at concentrations both above and below the MIC. We consider both biostatic antimicrobials, which decrease the division rate of microorganisms ($f_{S}^{\prime }<f_{S}$), and biocidal antimicrobials, which increase the death rate of microorganisms ($g_{S}^{\prime }>g_{S}$) [18].

We start from a microbial population where all individuals are S (sensitive), without antimicrobial. Specifically, we generally start our simulations with 10 S microorganisms, thus including a phase of initial growth, which can model the development of an infection starting from the bottleneck at transmission [35]. Our results are robust to variations of this initial condition, since we mainly consider timescales longer than that of the initial growth of the population to its equilibrium size. Note however that if we started with a very small number of S microorganisms (i.e. 1 or 2), we would need to take into account rapid stochastic extinctions (see S1 Appendix, Fig. IIIB).

Antimicrobial both drives the decrease of the population of sensitive microorganisms and selects for resistance. We ask whether resistance fully evolves *de novo*, leading to the C microorganisms taking over, or whether the microbial population goes extinct before this happens. The first case corresponds to treatment failure, and the second to treatment success. Hence, we are interested in the probability *p*_0_ of extinction of the microbial population before C microorganisms fix in the population, i.e. take over. We also discuss the average time *t*_*fix*_ it takes for the population to fully evolve resistance, up to full fixation of the C microorganisms, and the mean time to extinction before the fixation of the C type *t*_*ext*_.

We present both analytical and numerical results. Our analytical results are obtained using methods from stochastic processes, including the Moran process at fixed population size [13] and birth-death processes with time varying rates [36–39]. Our simulations employ a Gillespie algorithm [40, 41], and incorporate all individual stochastic division, mutation and death events with their exact rates (see S1 Appendix, section 5 for details).

## Results

### Conditions for a periodic presence of perfect biostatic antimicrobial to eradicate the microbial population

**Fig 1:**
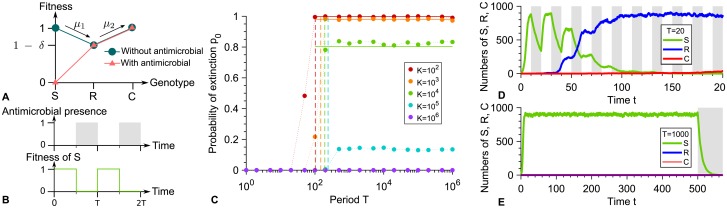
Periodic presence of a perfect biostatic antimicrobial. **A:** Microbial fitness versus genotype with and without antimicrobial. Genotypes are the following: S: sensitive; R: resistant; C: resistant-compensated. *δ* represents the fitness cost of resistance. **B:** Periodic presence of antimicrobial (gray: presence, white: absence), and impact on the fitness of S microorganisms. **C:** Probability *p*_0_ that the microbial population goes extinct before resistance gets established versus alternation period *T*, for various carrying capacities *K*. Markers: simulation results, with probabilities estimated over 10^2^ − 10^3^ realizations. Horizontal solid lines: analytical predictions from Eq 1. Dashed lines: *T*/2 = *τ*_*S*_. **D and E:** Numbers of S, R and C microorganisms versus time in example simulation runs for *K* = 1000, with *T* = 20 and *T* = 1000 respectively. In **D**, resistance takes over, while in **E**, extinction occurs shortly after antimicrobial is first added. Phases without (resp. with) antimicrobial are shaded in white (resp. gray). Parameter values: *f*_*S*_ = 1 without antimicrobial, $f_{S}^{\prime }=0$ with antimicrobial, *f*_*R*_ = 0.9, *f*_*C*_ = 1, *g*_*S*_ = *g*_*R*_ = *g*_*C*_ = 0.1, *μ*_1_ = 10^−5^ and *μ*_2_ = 10^−3^. All simulations start with 10 S microorganisms.

Do periodic alternations of phases with and without antimicrobial allow the eradication of a microbial population, or does resistance develop? We first address this question in the case of a biostatic antimicrobial sufficiently above the MIC to completely stop the growth of S microorganisms (see Fig 1A and 1B). With such a “perfect” biostatic antimicrobial, the fitness of S microorganisms is $f_{S}^{\prime }=0$, while without antimicrobial, *f*_*S*_ = 1. Here, we assume that the death rate of S microorganisms is not affected by the antimicrobial, i.e. $g_{S}^{\prime }=g_{S}$, but the case of a biocidal antimicrobial will be considered next. Note that within our logistic growth model, we consider that S microorganisms that cannot divide still consume resources, e.g. nutrients, in order to self-maintain. They may also still grow in size even if they cannot divide [3].

A crucial point is how the duration of a phase with antimicrobial, which corresponds here to the half-period *T*/2 of alternations, compares to the average time *τ*_*S*_ needed for a population of S microorganisms to go extinct in the presence of antimicrobial. Indeed, if *T*/2 ≫ *τ*_*S*_, one single phase with antimicrobial suffices to eradicate a microbial population in the absence of resistance. An exact first passage time calculation [33, 42] (see S1 Appendix, section 1.2, Eq. S7) yields $\tau_{S}=\left(1/g_{S}\right)\times \sum_{i=1}^{N}\left(1/i\right)\approx \log\left(N\right)/g_{S}$, where *N* ≫ 1 represents the number of microorganisms when antimicrobial is first added, i.e. at *T*/2. If the phase before antimicrobial is added is much longer than the initial growth timescale of the population, i.e. if *T*/2 ≫ 1/(*f*_*S*_ − *g*_*S*_) (see S1 Appendix, section 1.3.1), *N* can be taken equal to the deterministic equilibrium population size *N* = *K*(1 − *g*_*S*_/*f*_*S*_), obtained by setting the birth rate *f*_*S*_(1 − *N*/*K*) equal to the death rate *g*_*S*_. Hence, *τ*_*S*_ ≈ log[*K*(1 − *g*_*S*_/*f*_*S*_)]/*g*_*S*_. Note that in this regime, the initial population size has no impact on *τ*_*S*_, and that the division and death rates are both given by *g*_*S*_. Our simulation results in Fig 1C display an abrupt increase in the probability *p*_0_ that the microbial population goes extinct before developing resistance for *T* = 2*τ*_*S*_, in good agreement with our analytical prediction.

For fast alternations satisfying *T*/2 ≪ *τ*_*S*_, the phases with antimicrobial are not long enough to eradicate the microbial population, yielding a *systematic evolution of resistance*, and thus a vanishing probability *p*_0_ of extinction before resistance takes over. This prediction is confirmed by our simulation results in Fig 1C, and an example of resistance evolution in this regime is shown in Fig 1D. In the limit of very fast alternations, we expect an effective averaging of the fitness of S microorganisms, with ${\tilde{f}}_{S}=0.5$. Thus, an R mutant whose lineage will take over the population (i.e. fix) appears after an average time ${\tilde{t}}_{R}^{a}=1/\left(\tilde{N}\mu_{1}g_{S}{\tilde{p}}_{SR}\right)$ where $\tilde{N}\mu_{1}g_{S}$ represents the total mutation rate in the population, with $\tilde{N}=K\left(1-g_{S}/{\tilde{f}}_{S}\right)$, and where ${\tilde{p}}_{SR}=\left(1-{\tilde{f}}_{S}/f_{R}\right)/\left[1-{\left({\tilde{f}}_{S}/f_{R}\right)}^{\tilde{N}}\right]$ is the probability that a single R mutant fixes in a population of S microorganisms with constant size $\tilde{N}$, calculated within the Moran model [13]. Note that when the effective fitness of S microorganisms is ${\tilde{f}}_{S}$, acquiring resistance is beneficial (provided that the fitness cost of resistance is reasonable, namely smaller than 0.5). Subsequently, C mutants will appear and fix, thus leading to the full evolution of resistance in the population. The corresponding average total time *t*_*fix*_ of resistance evolution [33] obtained in our simulations agrees well with the analytical expression of ${\tilde{t}}_{R}^{a}$ for *T*/2 ≪ *τ*_*S*_ (see S1 Appendix, Fig. IVC).

Conversely, if *T*/2 ≫ *τ*_*S*_, *the microbial population is eradicated by the first phase with antimicrobial, provided that no resistant mutant preexists when antimicrobial is added to the environment*. Indeed, resistance cannot appear in the presence of a perfect biostatic antimicrobial since S microorganisms then cannot divide. Thus, in the absence of existing R mutants, extinction occurs shortly after time *T*/2 (see S1 Appendix, Fig. IVB), and the situation is equivalent to adding antimicrobial at *T*/2 and leaving it thereafter, as exemplified by Fig 1E. Hence, while they are longer than those usually encountered in periodic treatments, the longest periods considered here are relevant to describe extended continuous treatments. Note that although unlikely, fixation of resistance in the absence of antimicrobial will end up happening by spontaneous fitness valley crossing if the first phase without antimicrobial is long enough. Specifically, this will occur if *T*/2 ≫ *τ*_*V*_, where *τ*_*V*_ ≈ (*f*_*S*_ − *f*_*R*_)/(*μ*_1_ *μ*_2_ *g*_*S*_) is the average valley crossing time by tunneling, which is the relevant process unless populations are very small [17, 33, 43, 44]. Accordingly, our simulation results in S1 Appendix, Fig. IV, which includes longer alternation periods than Fig 1, feature three distinct regimes, and vanishing extinction probabilities are obtained for *T*/2 ≫ *τ*_*V*_, as well as for *T*/2 ≪ *τ*_*S*_.

Let us now focus on the regime where antimicrobial treatment can induce extinction of the microbial population, namely *τ*_*S*_ ≪ *T*/2 ≪ *τ*_*V*_, and calculate the extinction probability *p*_0_. A necessary condition for the population to be rescued by resistance [21] and avoid extinction is that at least one R mutant be present when antimicrobial is added. In the rare mutation regime *Kμ*_1_ ≪ 1, this occurs with probability $p_{R}=\tau_{R}^{d}/t_{R}^{app}=N\mu_{1}g_{S}\tau_{R}^{d}$, where $t_{R}^{app}=1/\left(N\mu_{1}g_{S}\right)$ is the average time of appearance of a resistant mutant, while $\tau_{R}^{d}$ is the average lifetime of a resistant lineage (destined for extinction without antimicrobial), both calculated in a population of S individuals with fixed size *N* = *K*(1 − *g*_*S*_/*f*_*S*_) [13, 33]. Importantly, the presence of R mutants does not guarantee the rescue of the microbial population, because small subpopulations of R microorganisms may undergo a rapid stochastic extinction. The probability $p_{R}^{e}\left(i\right)$ of such an extinction event depends on the number of R microorganisms present when antimicrobial is added, which is *i* with a probability denoted by $p_{R}^{c}\left(i\right)$, provided that at least one R mutant is present. The probability *p*_0_ that the microbial population is not rescued by resistance and goes extinct can then be expressed as:

$$\begin{array}{c} p_{0}=1-p_{R}\sum_{i=1}^{N-1}p_{R}^{c}\left(i\right)\left(1-p_{R}^{e}\left(i\right)\right)\;. \end{array}$$ (1)

The probability $p_{R}^{c}\left(i\right)$ can be calculated within the Moran model since the population size is stable around *N* = *K*(1 − *g*_*S*_/*f*_*S*_) before antimicrobial is added. Specifically, it can be expressed as the ratio of the average time $\tau_{R,i}^{d}$ the lineage spends in the state where *i* mutants exist to the total lifetime $\tau_{R}^{d}$ of the lineage without antimicrobial: $p_{R}^{c}\left(i\right)=\tau_{R,i}^{d}/\tau_{R}^{d}$ (see S1 Appendix, section 3.1). Next, in order to calculate the probability $p_{R}^{e}\left(i\right)$ that the lineage of R mutants then quickly goes extinct, we approximate the reproduction rate of the R microorganisms by *f*_*R*_(1 − (*S*(*t*)+ *R*(*t*))/*K*)≈*f*_*R*_(1 − *S*(*t*)/*K*), where *S*(*t*) and *R*(*t*) are the numbers of S and R individuals at time *t*. Indeed, early extinctions of R mutants tend to happen shortly after the addition of antimicrobials, when *S*(*t*)≫*R*(*t*). Thus motivated, we further take the deterministic approximation $S(t)=K(1-g_{S}/f_{S})e^{-g_{S}{}^{t}}$, while retaining a stochastic description for the R mutants [36, 37]. We then employ the probability generating function $\phi_{i}\left(z,t\right)=\sum_{j=0}^{\infty }z^{j}P\left(j,t|i,0\right)$, where *i* is the initial number of R microorganisms, which satisfies $p_{R}^{e}\left(i\right)=\lim_{t\to \infty }P\left(0,t|i,0\right)=\lim_{t\to \infty }\phi_{i}\left(0,t\right)$. Solving the partial differential equation governing the evolution of *ϕ*_*i*_(*z*, *t*) (see S1 Appendix, section 3.2) yields [38, 39]

$$\begin{array}{c} p_{R}^{e}\left(i\right)=\lim_{t\to \infty }{\left[\frac{g_{R}\int_{0}^{t}e^{\rho (u)}du}{1+g_{R}\int_{0}^{t}e^{\rho (u)}du}\right]}^{i}\;, \end{array}$$ (2)

with

$$\begin{array}{c} \rho \left(t\right)=\int_{0}^{t}[g_{R}-f_{R}(1-\frac{S(u)}{K})]du\;. \end{array}$$ (3)

Eq 1 then allows us to predict the probability that the microbial population goes extinct thanks to the first addition of antimicrobial. Fig 1C demonstrates a very good agreement between this analytical prediction and our simulation results in the rare mutation regime *Kμ*_1_ ≪ 1, and Fig. VIII in S1 Appendix further demonstrates good agreement for each separate term of Eq 1 in this regime. For larger populations, the probability that the microbial population is rescued by resistance increases, and the extinction probability tends to zero for frequent mutations *Kμ*_1_ ≫ 1 because R mutants are then always present in the population, in numbers that essentially ensure their survival (see Fig 1C). Note that in our simulations presented in Fig 1, we chose *μ*_1_ = 10^−5^ for tractability. With realistic bacterial mutation probabilities, namely *μ*_1_ ∼ 10^−10^[45], the rare mutation regime remains relevant for much larger populations.

### Biocidal antimicrobials and imperfect biostatic ones allow an extra mechanism of rescue by resistance

**Fig 2:**
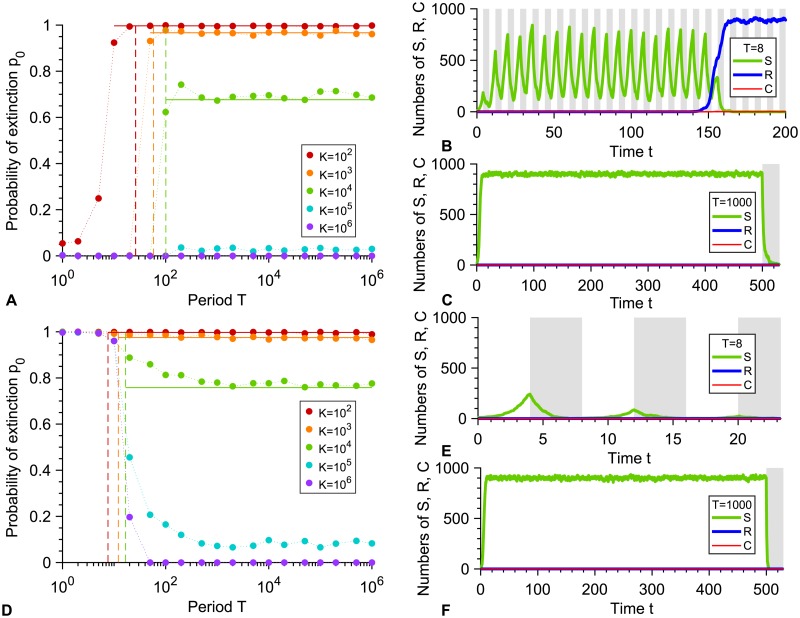
Periodic presence of a biocidal antimicrobial above the MIC. **A:** Probability *p*_0_ that the microbial population goes extinct before resistance gets established versus alternation period *T*, for various carrying capacities *K*. Markers: simulation results, with probabilities estimated over 10^2^ − 10^3^ realizations. Horizontal solid lines: analytical predictions from Eq 4. Dashed lines: *T*/2 = *τ*_*S*_. **B** and **C:** Numbers of sensitive (S), resistant (R) and compensated (C) microorganisms versus time in example simulation runs for *K* = 1000, with *T* = 8 and *T* = 1000 respectively. In **B**, resistance takes over, while in **C**, extinction occurs shortly after antimicrobial is first added. Phases without (resp. with) antimicrobial are shaded in white (resp. gray). Parameter values in **A**, **B** and **C**: *f*_*S*_ = 1, *f*_*R*_ = 0.9, *f*_*C*_ = 1, *g*_*S*_ = 0.1 without antimicrobial, $g_{S}^{\prime }=1.1$ with antimicrobial, *g*_*R*_ = *g*_*C*_ = 0.1, *μ*_1_ = 10^−5^ and *μ*_2_ = 10^−3^. All simulations start with 10 S microorganisms. **D**, **E** and **F**: same as **A**, **B** and **C**, but with $g_{S}^{\prime }=2$. All other parameters are the same.

How does the mode of action of the antimicrobial impact our results? So far, we considered a perfect biostatic antimicrobial that stops the growth of sensitive microorganisms but does not affect their death rate. Let us now turn to the general case of an antimicrobial that can affect both the division rate and the death rate of sensitive microorganisms, and let us assume that we are above the MIC, i.e. $g_{S}^{\prime }>f_{S}^{\prime }$. In this section, we present general calculations, but focus most of our discussion on purely biocidal antimicrobials, which increase the death rate of sensitive microorganisms without affecting their growth rate, and compare them to purely biostatic antimicrobials. Again, a crucial point is how the duration *T*/2 of a phase with antimicrobial compares to the average time *τ*_*S*_ needed for a population of S microorganisms to go extinct in the presence of antimicrobial (see Eq. S6). Indeed, our simulation results in Fig 2A and 2D display an abrupt change in the probability that the microbial population goes extinct before developing resistance for *T* = 2*τ*_*S*_.

For small periods *T*/2 ≪ *τ*_*S*_, one phase with antimicrobial is not long enough to eradicate the microbial population. However, *the alternations may induce an overall decrease in the population over multiple periods, then leading to extinction*. This is the case when the deterministic growth timescale 1/(*f*_*S*_ − *g*_*S*_) is larger than the decay timescale $1/(g_{S}^{\prime }-f_{S}^{\prime })$. Equivalently, in the limit of very fast alternations, there is no nonzero stationary population size when ${\tilde{f}}_{S}=\left(f_{S}+f_{S}^{\prime }\right)/2<{\tilde{g}}_{S}=\left(g_{S}+g_{S}^{\prime }\right)/2$, yielding the same condition. For a biostatic drug such that $g_{S}^{\prime }=g_{S}$, this situation cannot happen if *g*_*S*_ < *f*_*S*_/2, which is realistic since baseline death rates are usually small. Conversely, for a biocidal drug such that $f_{S}^{\prime }=f_{S}$, a systematic evolution of resistance will occur if $g_{S}^{\prime }<2f_{S}-g_{S}$, while population decay over several periods and extinction will occur if $g_{S}^{\prime }>2f_{S}-g_{S}$. These predictions are confirmed by the simulation results in Fig 2A and 2D, respectively, and the two different cases are exemplified in Fig 2B and 2E. Both of these regimes can arise, depending on the concentration of biocidal antimicrobial. Fig 2A–2C corresponds to concentrations just above the MIC, while Fig 2D–2F correspond to larger concentrations of bactericidal drugs, which can induce death rates equal to several times the birth rate [46, 47]. Note that in Fig 2A, the extinction probability is not zero for small periods with *K* = 10^2^: this is because stochastic extinctions can occur before resistance takes over for such a small equilibrium population size.

**Fig 3:**
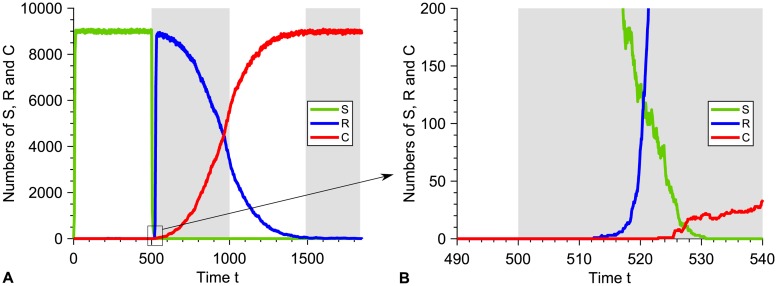
Resistance emergence in the presence of a biocidal antimicrobial above the MIC. **A:** Numbers of sensitive (S), resistant (R) and compensated (C) microorganisms versus time in an example simulation run for *K* = 10^4^, with *T* = 1000. Here resistance takes over. Phases without (resp. with) antimicrobial are shaded in white (resp. gray). **B:** Zoom showing the emergence of resistance in this realization: an R mutant appears after antimicrobial is added (gray). At this time, the S population is decreasing due to the antimicrobial-induced high death rate, but the surviving S microorganisms are still able to divide. Parameter values and initial conditions are the same as in Fig 2A, 2B and 2C.

For slower alternations satisfying *T*/2 ≫ *τ*_*S*_, *the microbial population is eradicated by the first phase with antimicrobial, unless resistance rescues it*. Extinction then occurs shortly after time *T*/2 (see S1 Appendix, Fig. VB and examples in Fig 2C and 2F). Importantly, with a biocidal antimicrobial or with an imperfect biostatic one, *the microbial population can be rescued by resistance in two different ways: either if resistant bacteria are present when antimicrobial is added, or if they appear afterwards*. This second case is exemplified in Fig 3. It can happen because even at high concentration, such antimicrobials do not prevent S microorganisms from dividing, contrarily to a perfect biostatic one. Because of this, rescue by resistance can become more likely than with perfect biostatic antimicrobials. Note that, as in the perfect biostatic case, the spontaneous fixation of resistant mutants without antimicrobial will occur if *T*/2 ≫ *τ*_*V*_ ≈ (*f*_*S*_ − *f*_*R*_)/(*μ*_1_ *μ*_2_ *g*_*S*_) (see S1 Appendix, Fig. V).

Let us focus on the regime where the treatment can efficiently induce extinction, namely *τ*_*S*_ ≪ *T*/2 ≪ *τ*_*V*_. The probability *p*_0_ that the microbial population is not rescued by resistance and goes extinct can then be expressed as:

$$\begin{array}{c} p_{0}=[1-p_{R}\sum_{i=1}^{N-1}p_{R}^{c}\left(i\right)\left(1-p_{R}^{e}\left(i\right)\right)][1-p_{R}^{a}\left(1-p_{R}^{e^{\prime }}\right)]\;. \end{array}$$ (4)

Apart from the last term, which corresponds to resistance appearing after antimicrobial is first added, Eq 4 is identical to Eq 1. The quantities *p*_*R*_ and $p_{R}^{c}\left(i\right)$ are the same as in that case, since they only depend on what happens just before antimicrobial is added. While $p_{R}^{e}\left(i\right)$ is conceptually similar to the perfect biostatic case, it depends on $f_{S}^{\prime }$ and $g_{S}^{\prime }$, and its general calculation is presented in Section 3.2 of the S1 Appendix. This leaves us with the new case where resistance appears in the presence of antimicrobial. In the rare mutation regime such that *N*_*div*_ *μ*_1_ ≪ 1, it happens with probability $p_{R}^{a}=N_{div}\mu_{1}$, where

$$\begin{array}{c} N_{div}=\int_{0}^{\tau_{S}}f_{S}(1-\frac{S(t)}{K})S\left(t\right)\;dt \end{array}$$ (5)

is the number of divisions that would occur in a population of S microorganisms between the addition of antimicrobial (taken as the origin of time here) and extinction. Employing the deterministic approximation for the number *S*(*t*) of S microorganisms (see Eq. S21), the probability that the lineage of an R mutant that appears at time *t*_0_ quickly goes extinct can be obtained in a similar way as for Eq 2, yielding

$$\begin{array}{c} p_{R}^{e^{\prime }}\left(t_{0}\right)=\lim_{t\to \infty }\frac{g_{R}\int_{t_{0}}^{t}e^{\eta (u)}du}{1+g_{R}\int_{t_{0}}^{t}e^{\eta (u)}du}\;, \end{array}$$ (6)

with

$$\begin{array}{c} \eta \left(t\right)=\int_{t_{0}}^{t}[g_{R}-f_{R}(1-\frac{S(u)}{K})]du\;. \end{array}$$ (7)

We then estimate the probability $p_{R}^{e^{\prime }}$ that the lineage of an R mutant that appears after the addition of antimicrobial quickly goes extinct by averaging $p_{R}^{e^{\prime }}\left(t_{0}\right)$ over the time *t*_0_ of appearance of the mutant, under the assumption that exactly one R mutant appears:

$$\begin{array}{c} p_{R}^{e^{\prime }}=\int_{0}^{\infty }p_{R}^{e^{\prime }}\left(t_{0}\right)\;\;{℘}_{R}^{a}\left(t_{0}\right)\;dt_{0}\;, \end{array}$$ (8)

with

$$\begin{array}{c} {℘}_{R}^{a}\left(t_{0}\right)=\frac{S\left(t_{0}\right)(1-\frac{S(t_{0})}{K})}{\int_{0}^{\infty }S\left(t\right)(1-\frac{S(t)}{K})dt}\;. \end{array}$$ (9)

Eq 4 then yields the probability that the microbial population goes extinct thanks to the first addition of antimicrobial. Fig 2A demonstrates a very good agreement between this analytical prediction and our simulation results in the rare mutation regime *Kμ*_1_ ≪ 1, and Figs. VIIIA-B, IX and X in S1 Appendix further demonstrate good agreement for each term involved in Eq 4 in this regime.

**Fig 4:**
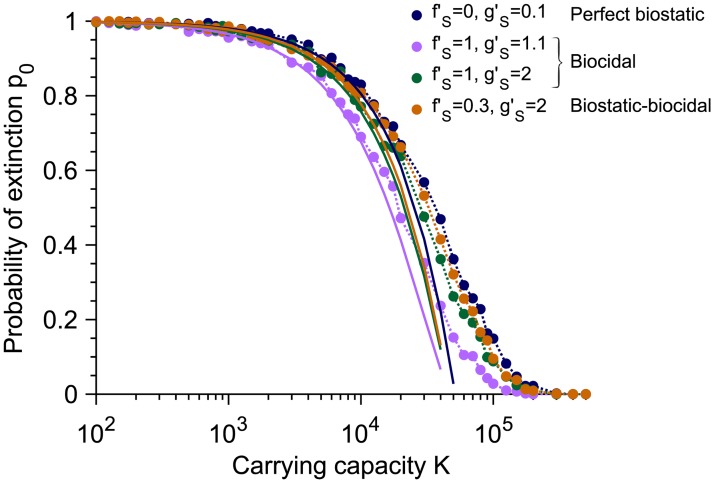
Dependence of the extinction probability *p*_0_ on population size and antimicrobial mode of action. The extinction probability *p*_0_ is plotted versus carrying capacity *K* for the perfect biostatic drug (corresponding to Fig 1), two different concentrations of biocidal drugs yielding two different death rates $g_{S}^{\prime }$ (corresponding to Fig 2) and a drug with both biostatic and biocidal effects. Markers correspond to simulation results, computed over 10^3^ realizations. Solid lines correspond to our analytical predictions from Eqs 1 and 4, respectively, which hold for *K* ≪ 1/*μ*_1_. Parameter values and initial conditions are the same as in Figs 1 and 2, respectively, and the period of alternations is *T* = 10^3^, which is in the large-period regime.

The extinction probability *p*_0_ depends on the size of the microbial population through its carrying capacity *K* and on the division and death rates with antimicrobial. Fig 4 shows the decrease of *p*_0_ with *K*, with *p*_0_ reaching 0 for *Kμ*_1_ ≫ 1 since resistant mutants are then always present when antimicrobial is added. Moreover, Fig 4 shows that *p*_0_ *depends on the antimicrobial mode of action*, with large death rates favoring larger *p*_0_ in the biocidal case, and with the perfect biostatic antimicrobial yielding the largest *p*_0_. Qualitatively, the observed increase of *p*_0_ as $g_{S}^{\prime }$ increases with a biocidal drug mainly arises from the faster decay of the population of S microorganisms, which reduces the probability $p_{R}^{a}$ that an R mutant appears in the presence of antimicrobial. Furthermore, one can show that the extinction probability *p*_0_ is larger for a perfect biostatic antimicrobial than for a perfect biocidal antimicrobial with $g_{S}^{\prime }\to \infty$ (see S1 Appendix, Section 3.4). Indeed, S microorganisms survive longer in the presence of a perfect biostatic drug, which reduces the division rate of the R mutants due to the logistic growth term, and thus favors their extinction. Such a competition effect is realistic if S microorganisms still take up resources (e.g. nutrients) even while they are not dividing. Besides, a treatment combining biostatic and biocidal effects yields a larger *p*_0_ than a pure biocidal one inducing the same death rate, thereby illustrating the interest of the additional biostatic effect (see Fig 4). Note that conversely, adding a biocidal to a perfect biostatic slightly decreases *p*_0_ due to the competition effect, as S microorganisms go extinct faster than with the perfect biostatic drug alone.

### Sub-MIC drug concentrations and stochastic extinctions

So far, we considered antimicrobial drugs above the MIC, allowing deterministic extinction in the absence of resistance for long enough drug exposure times. However, sub-MIC drugs can also have a major impact on the evolution of resistance, by selecting for resistance without killing large microbial populations, and moreover by facilitating stochastic extinctions in finite-sized microbial populations [18–20]. In the sub-MIC regime where $f_{S}^{\prime }>g_{S}^{\prime }$, the population has a nonzero deterministic equilibrium size $N^{\prime }=K\left(1-g_{S}^{\prime }/f_{S}^{\prime }\right)$ in the presence of antimicrobial. Nevertheless, stochastic extinctions can remain relatively fast, especially in the weakly-sub-MIC regime where $f_{S}^{\prime }$ is close to $g_{S}^{\prime }$, and if *K* is not very large. The key point is whether resistance appears before the extinction time *τ*_*S*_. The average time of appearance of an R mutant that fixes in a population of *N*′ individuals in the presence of sub-MIC antimicrobial is $t_{R}^{a}=1/\left(N^{\prime }\mu_{1}g_{S}^{\prime }p_{SR}^{\prime }\right)$, where $p_{SR}^{\prime }=\left[1-f_{S}^{\prime }g_{R}/\left(f_{R}g_{S}^{\prime }\right)\right]/\left[1-{\left(f_{S}^{\prime }g_{R}/\left(f_{R}g_{S}^{\prime }\right)\right)}^{N^{\prime }}\right]$ is the fixation probability of an R mutant in a population of S individuals with fixed size *N*′ (see S1 Appendix, Section 4, and Ref. [48]). Therefore, we expect resistance to take over and the extinction probability *p*_0_ to be very small if $t_{R}^{a}⪡\tau_{S}$ below the MIC, even for large periods such that *τ*_*S*_ < *T*/2.

**Fig 5:**
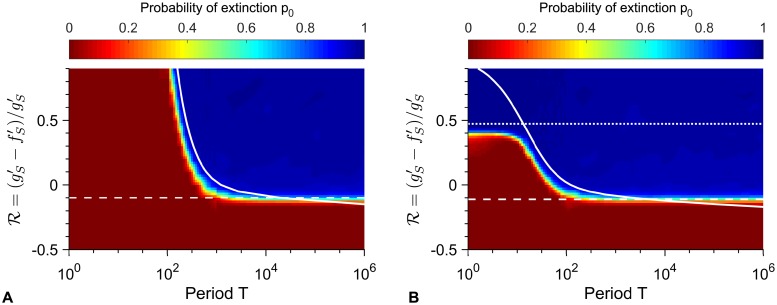
Heatmaps of the extinction probability. Extinction probability *p*_0_ versus alternation period *T* and $R=\left(g_{S}^{\prime }-f_{S}^{\prime }\right)/g_{S}^{\prime }$ with biostatic (**A**) or biocidal (**B**) antimicrobial. Heatmap: simulation data, each point computed over 10^3^ realizations of simulation results, and linearly interpolated. Dashed white line: value of R such that $t_{R}^{a}=\tau_{S}$ (see main text). Solid white line: *T*/2 = *τ*_*S*_. Parameter values: *K* = 10^3^, *μ*_1_ = 10^−5^, *μ*_2_ = 10^−3^, *f*_*S*_ = 1, *f*_*R*_ = 0.9, *f*_*C*_ = 1, *g*_*S*_ = *g*_*R*_ = *g*_*C*_ = 0.1, and (**A**) $g_{S}^{\prime }=0.1$ and variable $f_{S}^{\prime }$ or (**B**) $f_{S}^{\prime }=1$ and variable $g_{S}^{\prime }$. Dotted line in **B**: $R=\left(f_{S}-g_{S}\right)/\left(2f_{S}-g_{S}\right)$. All simulations start with 10 S microorganisms.

Fig 5 shows heatmaps of the probability *p*_0_ that the microbial population goes extinct before resistance takes over, in the cases of biostatic and biocidal drugs, plotted versus the period of alternations *T* and the non-dimensional variable $R=\left(g_{S}^{\prime }-f_{S}^{\prime }\right)/g_{S}^{\prime }$, which increases with antimicrobial concentration and is zero at the minimum inhibitory concentration (MIC). In both cases, two main regions are apparent, one with *p*_0_ = 0 and one where *p*_0_ is close to one. The transition between them is well described by the solid line *T*/2 = *τ*_*S*_ such that the time spent with drug is equal to the extinction time *τ*_*S*_ of a population of sensitive microbes with drug, except for large periods, where the relevant transition occurs below the MIC (R<0) and is given by $t_{R}^{a}=\tau_{S}$ (dashed line), consistently with our analytical predictions.

The ratio R enables us to make a quantitative comparison between biostatic and biocidal drugs. Let us focus first on the transition $\tau_{S}=t_{R}^{a}$. Eq. S6 shows that the average time it takes for the sensitive microorganisms to spontaneously go extinct in the presence of antimicrobial can be written as $\tau_{S}\left(f_{S}^{\prime },g_{S}^{\prime }\right)=\Phi \left(R\right)/g_{S}^{\prime }$, where *Φ* is a non-dimensional function. Besides, the average fixation time of a R mutant in a population of S individuals can also be expressed as $t_{R}^{a}\left(f_{S}^{\prime },g_{S}^{\prime }\right)=\Psi \left(R\right)/g_{S}^{\prime }$, where *Ψ* is a non-dimensional function. Thus, the transition $\tau_{S}=t_{R}^{a}$ will be the same for biostatic and biocidal drugs at a given value of R. Conversely, the transition *τ*_*S*_ = *T*/2, i.e. $\Phi \left(R\right)/g_{S}^{\prime }=T/2$, depends on $g_{S}^{\prime }$, and is thus different for biostatic and biocidal drugs at the same value of R. Specifically, for a given value of R, smaller periods *T* will suffice to get extinction after the first addition of antimicrobial for a biocidal drug than for a biostatic drug, because $g_{S}^{\prime }$ is increased by biocidal drugs, and hence *τ*_*S*_ is smaller in the biocidal case than in the biostatic case. This means that *the parameter regime where treatment is efficient is larger for biocidal drugs than for biostatic drugs, as can be seen by comparing* Fig 5A and 5B. Significantly above the MIC, another difference is that *biocidal drugs become efficient even for short periods T*/2 ≪ *τ*_*S*_ *if their concentration is large enough to have* $g_{S}^{\prime }>2f_{S}-g_{S}$, i.e. $R>\left(f_{S}-g_{S}\right)/\left(2f_{S}-g_{S}\right)$ (see above, esp. Fig 2D and 2E). Numerical simulation results agree well with this prediction (dotted line on Fig 5B).

**Fig 6:**
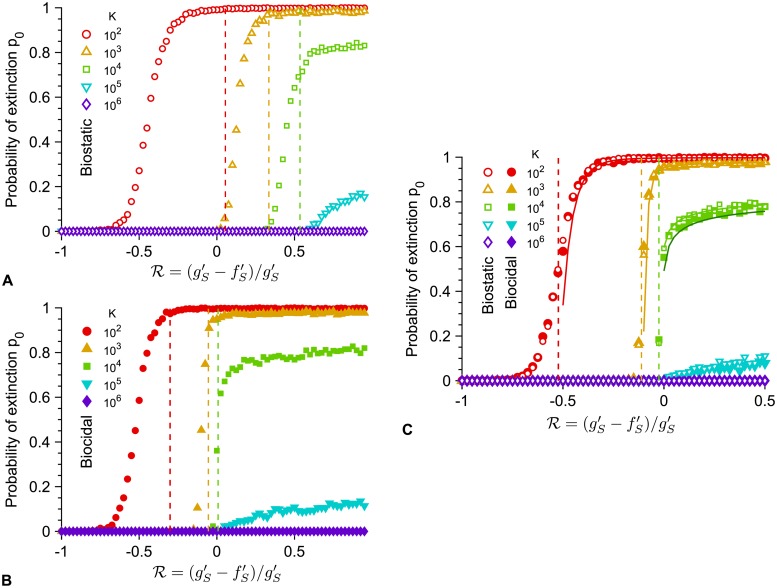
Dependence of the extinction transition on population size and antimicrobial mode of action. Extinction probability *p*_0_ versus the ratio $R=\left(g_{S}^{\prime }-f_{S}^{\prime }\right)/g_{S}^{\prime }$ with biostatic or biocidal antimicrobial, for different carrying capacities *K*, either in the small-period regime, with *T* = 10^2.5^ (**A** and **B**) or in the large-period regime, with *T* = 10^5^ (**C**). Markers: simulation results, calculated over 10^3^ realizations. Vertical dashed lines: predicted extinction thresholds, i.e. values of R such that *T*/2 = *τ*_*S*_ (**A** and **B**) or $t_{R}^{a}=\tau_{S}$ (**C**). Solid lines (**C**): Analytical estimates of *p*_0_ from Eq 1 (biostatic) or Eq 4 (biocidal). For *K* = 10^2^ and 10^3^, the analytical predictions in the biostatic and biocidal case are confounded, while for *K* = 10^4^ we used two shades of green to show the slight difference (light: biostatic, dark: biocidal). Parameter values: *μ*_1_ = 10^−5^, *μ*_2_ = 10^−3^, *f*_*S*_ = 1, *f*_*R*_ = 0.9, *f*_*C*_ = 1, *g*_*S*_ = *g*_*R*_ = *g*_*C*_ = 0.1, and $g_{S}^{\prime }=0.1$ (biostatic) or $f_{S}^{\prime }=1$ (biocidal). All simulations start with 10 S microorganisms.

Importantly, *the transition between large and small extinction probability when R (and thus the antimicrobial concentration) is varied strongly depends on population size, specifically on carrying capacity* (Fig 6 and S1 Appendix, *Fig. VI*), *and also depends on antimicrobial mode of action* (Fig 6). For small periods where the relevant transition occurs for *T*/2 = *τ*_*S*_, concentrations above the MIC (R>0) can actually be necessary to get extinction because one period may not suffice to get extinction, and moreover, the extinction threshold value R is not the same for biostatic and biocidal antimicrobials (see above and Fig 6A and 6B). Conversely, for large periods where the relevant transition occurs for $t_{R}^{a}=\tau_{S}$, and extinction occurs upon the first addition of drug, the extinction threshold is always below the MIC (R<0) and it is the same for biostatic and biocidal antimicrobials (see above and Fig 6C). In both cases, the larger the population, the larger the concentration required to get large extinction probabilities. For large periods (Fig 6C), the transition occurs close to the MIC for large populations, but the smaller the population, the larger the discrepancy between the MIC and the actual transition, as predicted by our analytical estimate based on $t_{R}^{a}=\tau_{S}$ (see S1 Appendix, Fig. VI). This is because in small populations, stochastic extinctions of the population are quite fast at weakly sub-MIC antimicrobial. This is a form of inoculum effect, where the effective MIC depends on the size of the bacterial population [20]. In the large period regime (Fig 6C), the extinction probability *p*_0_ is well-predicted by Eqs. 1 and 4 for the R values such at most one R mutant can appear before the extinction of the population (as assumed in our calculation of $p_{R}^{a}$). In this regime, the extinction time is close to *T*/2 (see S1 Appendix, Fig. VII) as extinction is due to the first addition of antimicrobial, while for smaller R values, extinction occurs after multiple periods.

In Fig 6A and 6B, transitions between small and large values of *p*_0_ in simulated data are observed for smaller threshold values of R than predicted by *T*/2 = *τ*_*S*_ (this can also be seen in Fig 5, where the solid white line is somewhat in the blue zone corresponding to large *p*_0_). This is because we have employed the average extinction time *τ*_*S*_, while extinction is a stochastic process. Thus, even if *T*/2 < *τ*_*S*_, upon each addition of antimicrobial, there is a nonzero probability that extinction actually occurs within the half-period. Denoting by *p* the probability that a given extinction time is smaller than *T*/2, the population will on average go extinct after 1/*p* periods, unless resistance fixes earlier. For instance, a population with carrying capacity *K* = 10^2^ submitted to alternations with *T* = 10^2.5^ is predicted to develop resistance before extinction if R<0.055. However, for R=-0.1, simulations yield a probability *p*_0_ = 0.99 of extinction before resistance takes over (see Fig 6A). In this case, simulations yield *p* = 0.3, implying that extinction typically occurs in ~3 periods, thus explaining the large value of *p*_0_. More generally, the probability distribution function of the extinction time can depend on various parameters, which can impact the discrepancy between the predicted and observed transitions. A more precise calculation would involve this distribution. Note that the distribution of extinction times is known to be exponential for populations with a quasi-stationary state [49, 50], but the present situation is more complex because there is no nonzero deterministic equilibrium population size below the MIC, and because the population size at the time when antimicrobial is added is far from the equilibrium value with antimicrobial. Nevertheless, our prediction based on the average extinction time *τ*_*S*_ yields the right transition shape (see Fig 5) and the correct expectations for *T*/2 ≫ *τ*_*S*_ and *T*/2 ≪ *τ*_*S*_.

## Discussion

### Main results

The evolution of antimicrobial resistance often occurs in variable environments, as antimicrobial is added and removed from a medium or given periodically to a patient, e.g. in a treatment by the oral route [3, 4]. Alternations of phases of absence and presence of antimicrobial induce a dramatic time variability of selection pressure on microorganisms, and can thus have a strong impact on resistance evolution. Using a general stochastic model which includes variations of both composition and size of the microbial population, we have shed light on the impact of periodic alternations of presence and absence of antimicrobial on the probability that resistance evolves *de novo* and rescues a microbial population from extinction. The majority of previous studies of periodic antimicrobial treatments [10, 51–56] neglect stochastic effects, while they can have a crucial evolutionary impact [13, 15], especially on population extinction [18, 20]. In addition, established microbial populations are structured, even within a single patient [57], and competition is local, which decreases their effective size, thus making stochasticity relevant. While a few previous studies did take stochasticity into account, some did not include logistic growth or compensation of the cost of resistance [36], while others made specific assumptions on treatments or epidemiology [58, 59], focused on numerical results with few analytical predictions [60], or assumed a constant population size [33]. The present model has the advantage of being quite general while fully accounting for stochasticity and finite-population effects.

We showed that fast alternations of presence and absence of antimicrobial are inefficient to eradicate the microbial population and strongly favor the establishment of resistance, unless the antimicrobial increases enough the death rate, which can occur for biocidal antimicrobials at high concentration [46, 47]. The corresponding criterion on the death rate $g_{S}^{\prime }$ of sensitive microorganisms with biocidal antimicrobial, namely $g_{S}^{\prime }>2f_{S}-g_{S}$, is generally more stringent than simply requiring drug concentrations to be above the MIC during the phases with biocidal antimicrobial, namely $g_{S}^{\prime }>f_{S}$. Indeed, the population can re-grow without antimicrobial: in this regime, extinction occurs over multiple periods, and involves decaying oscillations. Conversely, for biostatic antimicrobials, as well as for biocidal ones at smaller concentrations, extinction has to occur within a single phase with antimicrobial, and thus the half-period *T*/2 has to be longer than the average extinction time *τ*_*S*_, which we fully expressed analytically. Importantly, shorter periods suffice for biocidal antimicrobials compared to biostatic ones in order to drive a population to extinction upon the first addition of antimicrobial, at the same value of $R=\left(g_{S}^{\prime }-f_{S}^{\prime }\right)/g_{S}^{\prime }$. Hence, the parameter regime where treatment is efficient is larger for biocidal drugs than for biostatic drugs. If *T*/2 > *τ*_*S*_, the microbial population goes extinct upon the first addition of antimicrobial, unless it is rescued by resistance. We obtained an analytical expression for the probability *p*_0_ that the population is eradicated upon the first addition of antimicrobial, assuming rare mutations. Note that with realistic bacterial mutation probabilities, namely *μ*_1_ ∼ 10^−10^[45], the rare mutation regime remains relevant even for quite large populations. Moreover, real microbial populations are generally structured, which reduces their effective population size. Rescue by resistance can happen either if resistant mutants preexist upon the addition of antimicrobial, or if they appear after antimicrobial is added to the environment, during the decay of the population. Importantly, the latter case is fully prevented by perfect biostatic antimicrobials that completely stop division of sensitive microorganisms. This sheds light on the respective merits of different antimicrobial modes of action. Finally, we showed that due to stochastic extinctions, sub-MIC concentrations of antimicrobials can suffice to yield extinction of the population, and we fully quantified this effect and its dependence on population size. Throughout, all of our analytical predictions were tested by numerical simulations, and the latter also allowed us to explore cases beyond the rare mutation regime, where resistance occurs more frequently.

This work opens many possible theoretical extensions. In particular, it will be very interesting to include effects such as antibiotic tolerance, which tend to precede resistance under intermittent antibiotic exposure [4], as well as to consider the possibility of concentrations above the mutant prevention concentration, such that resistant microbes are also affected by the drug [4, 55]. Another exciting extension would be to incorporate spatial structure [61–63] and environment heterogeneity, in particular drug concentration gradients. Indeed, static gradients can strongly accelerate resistance evolution [64–67], and one may ask how this effect combines with the temporal alternation-driven one investigated here. Besides, it would be interesting to explicitly model horizontal gene transfer of resistance mutations, to include realistic pharmacodynamics and pharmacokinetics [10], and also to compare the impact of periodic alternations to that of random switches of the environment [5–9, 68–70]. Other effects such as single-cell physiological properties [3], phenotypic delay [71] or density dependence of drug efficacy [72] can further enrich the response of microbial populations to variable concentrations of antimicrobials.

### Practical relevance

Our results have consequences for actual experimental and clinical situations. First, several of our predictions can be tested experimentally in controlled setups such as that presented in Ref. [3]. This would allow for an experimental test of the transition of extinction probability between the short-period and the long-period regimes, and of the predicted values of this extinction probability for large periods in the rare mutation regime. Second, the situation where the phases of absence and presence of antimicrobial have similar durations, which we considered here, is unfortunately clinically realistic. Indeed, a goal in treatment design is that the serum concentration of antimicrobial exceeds the MIC for at least 40 to 50% of the time [11]. Because bacteria divide on a timescale of about an hour in exponential growth phase, and because antimicrobial is often taken every 8 to 12 hours in treatments by the oral route, the alternation period lasts for a few generations in treatments: this is the same order of magnitude as the transition we found between the short-period and long-period regimes, meaning that this transition is relevant in clinical cases. Note that while this transition timescale depends on the death and birth rates of sensitive microbes in the presence of antimicrobial (see Eq. S6), and therefore on antimicrobial concentration, it does not depend on the value of the mutation rate or on the initial population size (as long as the half-period is longer than the initial population growth timescale, see S1 Appendix, section 1.3.1), and it depends only weakly on the carrying capacity, e.g. logarithmically in the perfect biostatic case (see Eq. S7). Given the relevance of this transition between the short-period and the long-period regimes, it would be very interesting to conduct precise measurements of both division rates and death rates [73] in actual infections in order to determine the relevant regime in each case. This is all the more important that in the short-period regime, we showed that only large concentrations of biocidal antimicrobials are efficient, while other antimicrobials systematically lead to the *de novo* evolution of resistance before eradication of the microbial population. This constitutes a striking argument in favor of the development of extended-release antimicrobial formulations [74]. Conversely, a broader spectrum of modes of action can be successful for longer periods of alternation of drug absence and presence.

Despite the fact that only biocidal antimicrobials at high concentration are efficient for short alternation periods of absence and presence of drug, and the fact that the parameter regime where treatment is efficient is larger for biocidal drugs than for biostatic drugs, biostatic antimicrobials that fully stop division of sensitive microorganisms have a distinct advantage over drugs with other modes of action. Indeed, they prevent the emergence of resistant mutants when drug is present, which is all the more important that such resistant mutants are immediately selected for by the antimicrobial and are thus quite likely to rescue the microbial population and to lead to the fixation of resistance. This argues in favor of combination therapies involving a biostatic and a biocidal antimicrobial. Note however that the combined drugs need to be chosen carefully, because some of them have antagonistic interactions [75], depending on their mode of action.

## Supporting information

S1 Appendix Methodological details and further results. In S1 Appendix., we present additional details about our model and methods, as well as further results.
(PDF)
