# Supplementary material for: Resist or perish: Fate of a microbial population subjected to a periodic presence of antimicrobial
Source: PLoS Comput Biol. 2020 Apr 10;16(4):e1007798. doi: 10.1371/journal.pcbi.1007798 (PMC7176291; doi:10.1371/journal.pcbi.1007798)
Supplement: S1 Appendix — In S1 Appendix., we present additional details about our model and methods, as well as further results. (PDF) [file pcbi.1007798.s001.pdf]

Supporting information for “Resist or perish: fate of a microbial population subjected to a periodic presence of antimicrobial”

Loïc Marrec[1], Anne-Florence Bitbol[1,2*]

1 Sorbonne Université, CNRS, Institut de Biologie Paris-Seine, Laboratoire Jean Perrin (UMR 8237), F-75005 Paris, France
2 Institute of Bioengineering, School of Life Sciences, École Polytechnique Fédérale de Lausanne (EPFL), CH-1015 Lausanne, Switzerland

* anne-florence.bitbol@epfl.ch

Contents

1 Population with a single type of microorganisms 2
- 1.1 Master equation . . . 2
- 1.2 Average spontaneous extinction time . . . 2
- 1.3 Initial growth of the population . . . 4
  - 1.3.1 Deterministic approximation and rise time . . . 4
  - 1.3.2 Probability of rapid initial extinction . . . 4

2 Supplementary results on extinction probabilities and extinction and fixation times 6
- 2.1 Perfect biostatic antimicrobial . . . 6
- 2.2 Biocidal antimicrobial . . . 7
- 2.3 Population size dependence of the extinction transition . . . 8
- 2.4 Dependence of the extinction time on population size and antimicrobial mode of action . . . 9

3 Rescue by resistance 10
- 3.1 Number of resistant mutants when antimicrobial is added: $p_R^c(i)$ . . . 10
- 3.2 Probability of fast extinction of the resistant mutants: $p_R^e(i)$ . . . 10
- 3.3 Predicting the extinction probability p_0 . . . 12
- 3.4 A perfect biostatic antimicrobial yields a larger p_0 than a perfect biocidal antimicrobial . . . 15

4 Fixation probability of a mutant in a population of constant size 16

5 Detailed simulation methods 17

1 Population with a single type of microorganisms

1.1 Master equation

Let us first consider the simple case of a microbial population with a carrying capacity K comprising a single type of microorganisms. These microorganisms have a fitness and a death rate denoted by f and g, respectively. Let j be the number of individuals in the population at time t, satisfying $0 \leq j \leq K$. The master equation describing the evolution of this population reads for all j:

$$\frac{dP_j(t)}{dt} = f\left(1-\frac{j-1}{K}\right)(j-1)P_{j-1}(t) + g(j+1)P_{j+1}(t) - \left(f\left(1-\frac{j}{K}\right)+g\right)jP_j(t)\ . \tag{S1}$$

Indeed, recall that $f(1-j/K)$ is the division rate in the logistic model. We can write this system of equations as $\dot{\mathbf{P}} = \mathbf{RP}$, where $\mathbf{R}$ is the transition rate matrix:

$$\frac{d}{dt}\begin{pmatrix} P_0 \\ P_1 \\ P_2 \\ \vdots \\ P_K \end{pmatrix} = \begin{pmatrix} 0 & g & 0 & \cdots & 0 \\ 0 & -g-f(1-1/K) & 2g & (0) & \vdots \\ 0 & f(1-1/K) & -2g-2f(1-2/K) & \ddots & 0 \\ \vdots & (0) & \ddots & \ddots & Kg \\ 0 & \cdots & 0 & f(1-(K-1)/K)(K-1) & -Kg \end{pmatrix}\begin{pmatrix} P_0 \\ P_1 \\ P_2 \\ \vdots \\ P_K \end{pmatrix}\ . \tag{S2}$$

This Markov chain has a single absorbing state, namely $j = 0$, which corresponds to the extinction of the microbial population.

1.2 Average spontaneous extinction time

Let us study the average time it takes for the population to spontaneously go extinct, i.e. the mean first-passage time $\tau_S(j_0)$ to the absorbing state $j = 0$, starting from j_0 microorganisms at $t = 0$. It can be expressed using the inverse of the reduced transition rate matrix $\widetilde{\mathbf{R}}$, which is identical to $\mathbf{R}$ except that the row and the column corresponding to the absorbing state $j = 0$ are removed [1, 2]:

$$\tau_S(j_0) = \mathbb{E}[\widehat{\tau}_{FP}\,|\,j_0] = -\sum_{i=1}^{K}(\widetilde{\mathbf{R}}^{-1})_{i\,j_0}\ . \tag{S3}$$

Note that more generally, all the moments of the first-passage time can be obtained using the reduced transition rate matrix $\widetilde{\mathbf{R}}$:

$$\mathbb{E}[\widehat{\tau}_{FP}^{n}\,|\,j_0] = n!(-1)^n\sum_{i=1}^{K}(\widetilde{\mathbf{R}}^{-n})_{i\,j_0}\ . \tag{S4}$$

Here, the elements of the inverse of the reduced transition matrix read for all $1 \leq j \leq K$,

$$(\widetilde{\mathbf{R}}^{-1})_{i\,j} = \begin{cases} -\displaystyle\sum_{k=0}^{i-1}\left(\frac{f}{g}\right)^{i-k-1}\frac{K^{k+1-i}(K-k-1)!}{i\,g\,(K-i)!} & \text{if } i \leq j\ , \\ -\displaystyle\sum_{k=0}^{j-1}\left(\frac{f}{g}\right)^{i-k-1}\frac{K^{k+1-i}(K-k-1)!}{i\,g\,(K-i)!} & \text{if } i > j\ . \end{cases} \tag{S5}$$

Substituting Eq. S5 in Eq. S3 yields

$$\tau_S(j_0) = \frac{1}{g}\left[\sum_{i=1}^{j_0}\sum_{k=0}^{i-1}\left(\frac{f}{g}\right)^{i-k-1}\frac{K^{k+1-i}(K-k-1)!}{i\,(K-i)!} + \sum_{i=j_0+1}^{K}\sum_{k=0}^{j_0-1}\left(\frac{f}{g}\right)^{i-k-1}\frac{K^{k+1-i}(K-k-1)!}{i\,(K-i)!}\right]\ . \tag{S6}$$

If $f = 0$, e.g. in the presence of a biostatic antimicrobial that perfectly prevents all microorganisms from growing, Eq. S6 simplifies to:

$$\tau_S(j_0) = \frac{1}{g}\sum_{i=1}^{j_0}\frac{1}{i}\ . \tag{S7}$$

Note the formal analogy between Eq. S7 and the unconditional fixation time with biostatic antimicrobial ($f = 0$) in the Moran process, which corresponds to the extinction of the sensitive microbes in a population of fixed size [1]. Both situations involve the extinction of microorganisms that do not grow. Formally, the master equation of a Moran process describing a microbial population of fixed size N with two types of individuals A and B whose respective fitnesses are $f_A = 0$ and $f_B = 1$, reads:

$$\frac{dP_l(t)}{dt} = \frac{l+1}{N} P_{l+1}(t) - \frac{l}{N} P_l(t) \ , \tag{S8}$$

where l denotes the number of A individuals. The master equation for a logistic growth of a population with a single type of individuals (see Eq. S1) with $f = 0$ is equivalent under the transformation $1/N \leftarrow g$.

Fig. I shows how $\tau_S(10)$ depends on the death rate g and the carrying capacity K. In particular, it shows that when $g < f$, average extinction times become very long for large values of K, while they are short for all K when $g > f$. In a deterministic description (valid for very large population sizes), $g = f$ indeed corresponds to the transition between a population that decays exponentially and a population that reaches a steady state size. For finite-sized populations, stochasticity makes this transition smoother.

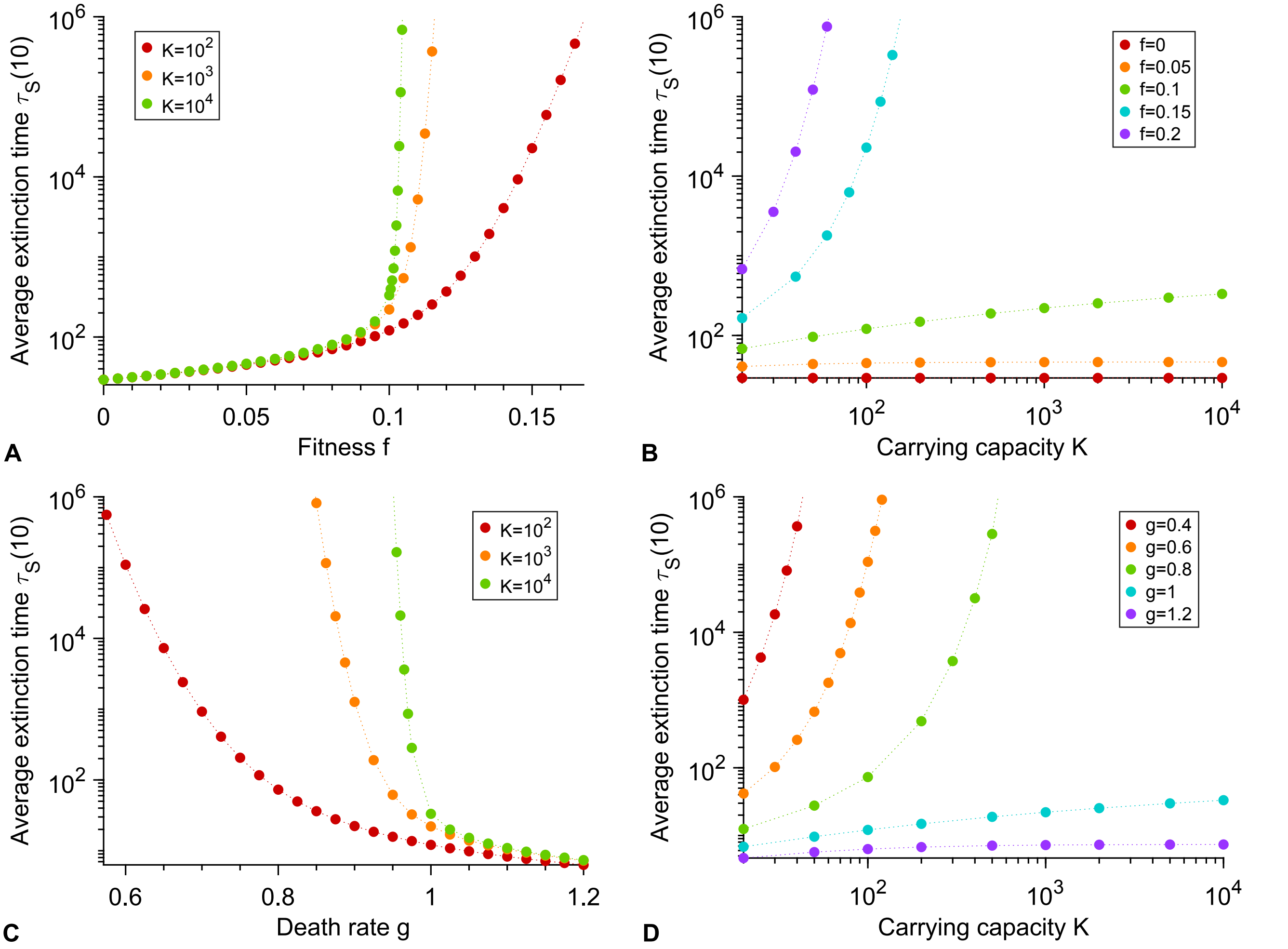

Fig I. Average spontaneous extinction time of the microbial population. A: Mean first-passage time $\tau_S(10)$ to the absorbing state $j = 0$, i.e. average extinction time, starting from $j_0 = 10$ microorganisms, as a function of the fitness f for different carrying capacities K, with $g = 0.1$. **B:** Average extinction time $\tau_S(10)$ as a function of the carrying capacity K for different fitnesses f, with $g = 0.1$. **C:** Average extinction time $\tau_S(10)$ as a function of the death rate g for different carrying capacities K, with $f = 1$. **D:** Average extinction time $\tau_S(10)$ as a function of the carrying capacity K for different death rates g, with $f = 1$.

1.3 Initial growth of the population

1.3.1 Deterministic approximation and rise time

In the deterministic regime, for a population with only one type of microorganisms and a carrying capacity K, the number N of individuals at time t follows the logistic ordinary differential equation:

$$\frac{dN(t)}{dt} = N(t)\left[f\left(1-\frac{N(t)}{K}\right)-g\right], \tag{S9}$$

where f represents fitness and g death rate. For $f \neq g$, the solution reads:

$$N(t) = \frac{K\,N_0\,e^{(f-g)t}\,(1-g/f)}{K\,(1-g/f)+N_0\,(e^{(f-g)t}-1)}, \tag{S10}$$

where $N_0 = N(0)$ is the initial number of individuals in the population. Note that we recover the usual law of logistic population growth for $f > 0$ and $g = 0$ (or for $f > g$ by setting $f \leftarrow f - g$):

$$N(t) = \frac{K\,N_0\,e^{f\,t}}{K+N_0\,(e^{f\,t}-1)}. \tag{S11}$$

For $f > g$, the long-time limit of Eq. S10 is $K(1-g/f)$. This equilibrium population size can also be found as the steady-state solution of Eq. S9, and corresponds to the birth and death rates being equal. The rise time $t_r(\alpha)$, at which a fraction α of this equilibrium population size is reached, is given by:

$$t_r(\alpha) = \frac{1}{f-g}\ln\left(\frac{\alpha K(1-g/f)-\alpha N_0}{(1-\alpha)N_0}\right). \tag{S12}$$

Hence, the initial growth of the population is governed by the timescale $1/(f-g)$, and features a weaker dependence on carrying capacity K and initial population size N_0, as illustrated by Fig. II.

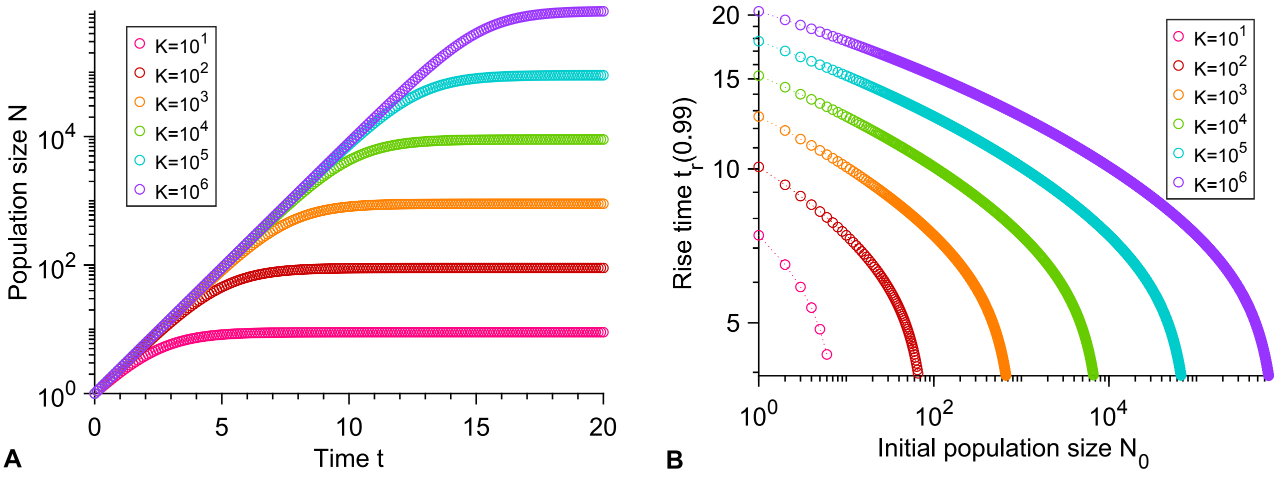

Fig II. Deterministic evolution of the population size and rise time. A: Population size N as function of time t for different carrying capacities K. **B:** rise time $t_r(0.99)$ as function of the initial number of individuals N_0 for different carrying capacities K. Results are obtained from Eqs. S10 and S12. Parameter values: $f = 1$ and $g = 0.1$.

1.3.2 Probability of rapid initial extinction

A microbial population starting with few individuals may go extinct quickly due to stochastic fluctuations, before reaching a substantial fraction of its equilibrium size $K(1-g/f)$. Formally integrating the master equation $\dot{\mathbf{P}} = \mathbf{RP}$ with the initial condition $j = j_0$ allows to express the probability $P_0(t)$ that a population starting from j_0 microorganisms at $t = 0$ is extinct at time t:

$$P_0(t) = (e^{\mathbf{R}t})_{0j_0}. \tag{S13}$$

Fig. III shows the probability $P_0(t_r)$ that the microbial population goes extinct before the rise time t_r versus g for $f = 1$. We notice that $P_0(t_r) \sim g/f$ for small g and/or large K. This result can be proved analytically by assuming that the number of individuals is very small compared to the carrying capacity K and thus grows exponentially, which is relevant when rapid initial extinctions occur. One can then neglect the impact of the carrying capacity K in the master equation Eq. S1, yielding:

$$\frac{dP_j(t)}{dt} = f(j-1)P_{j-1}(t) + g(j+1)P_{j+1}(t) - (f+g)\,jP_j(t)\ . \tag{S14}$$

The solution of this master equation is given by [3]:

$$P_j(t) = e^{(f-g)t}\left(\frac{1-g/f}{e^{(f-g)t}-g/f}\right)^2\left(\frac{e^{(f-g)t}-1}{e^{(f-g)t}-g/f}\right)^{j-1}\ . \tag{S15}$$

In particular, we thus obtain:

$$P_0(t) = \frac{g}{f}\left(\frac{e^{(f-g)t}-1}{e^{(f-g)t}-g/f}\right)\underset{t\to+\infty}{\to}\frac{g}{f}\ \text{if}\ f>g\ . \tag{S16}$$

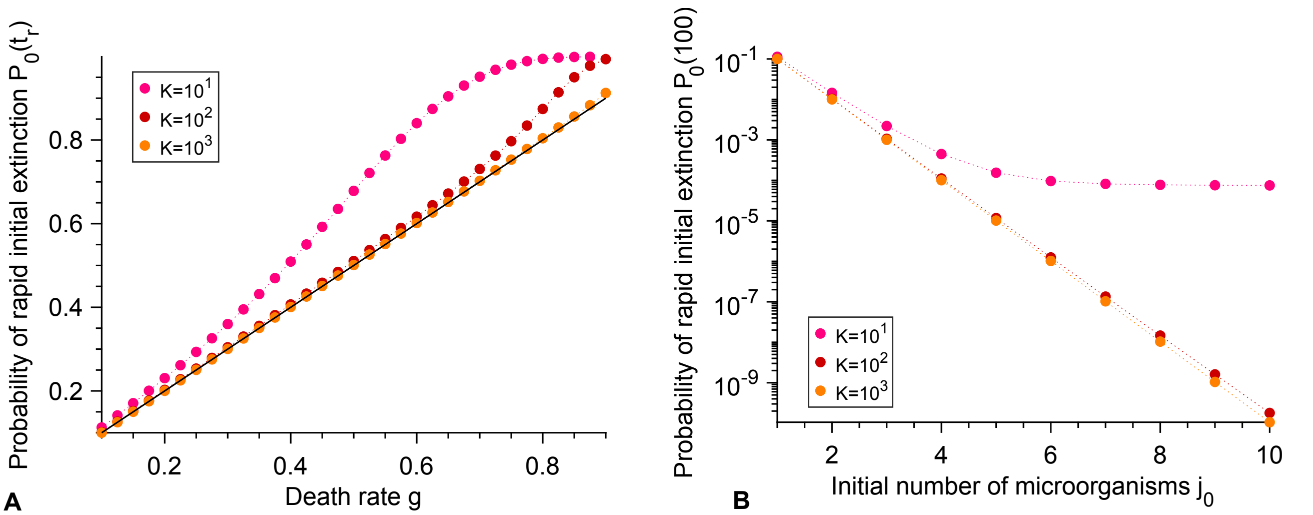

Fig III. Rapid initial extinction. **A:** Probability $P_0(t_r)$ that extinction occurs before the rise time $t_r(0.99)$ (see Eq. S12), when starting from a single microorganism, $j_0 = 1$, as function of the death rate g with $f = 1$ for different carrying capacities K. Results come from a numerical computation of Eq. S13. Solid black line: g/f. **B:** Probability of rapid initial extinction $P_0(100)$ as a function of the initial number of microorganisms j_0, for different carrying capacities K. Data points correspond to numerical computations of Eq. S13. Parameter values: $f_S = 1$ and $g_S = 0.1$. Time $t = 100$ was chosen to evaluate P_0 because it is larger than typical rise times for the parameter values considered (see Fig. II), but not too long, and thus captures rapid initial extinctions but not long-term ones (see Fig. I).

2 Supplementary results on extinction probabilities and extinction and fixation times

2.1 Perfect biostatic antimicrobial

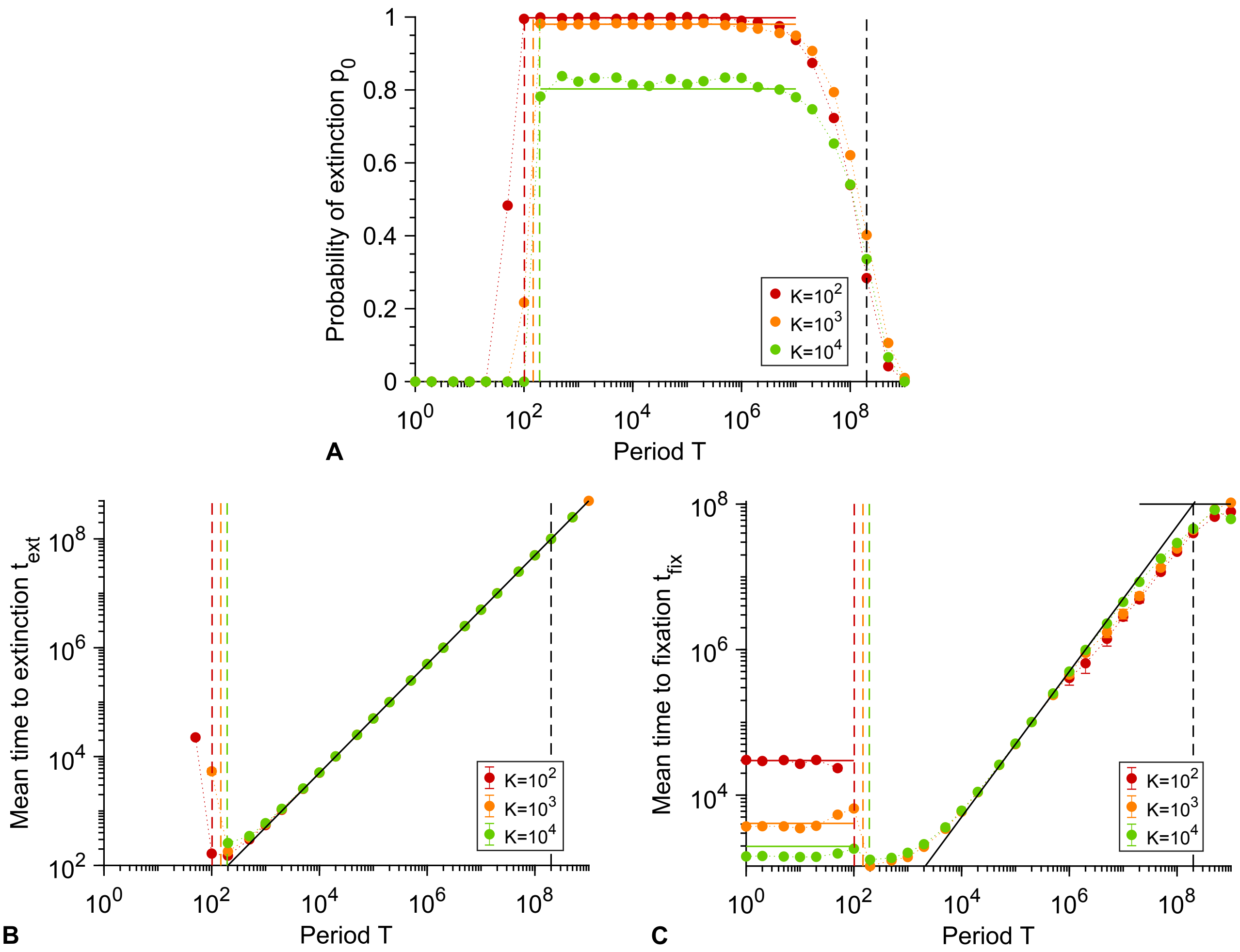

Fig IV. Periodic presence of a biostatic antimicrobial that fully stops growth, including long periods. A: Probability p_0 that the microbial population goes extinct before resistance gets established versus alternation period T, for various carrying capacities K. Markers: simulation results, with probabilities estimated over $10^2 - 10^3$ realizations. Horizontal solid colored lines: analytical predictions from Eq. 1. Horizontal solid black line: average spontaneous valley crossing time $\tau_V = (f_S - f_R)/(\mu_1 \mu_2 g_S)$ (see main text). **B:** Average time t_{ext} to extinction versus alternation period T for various carrying capacities K. Data shown if extinction occurred in at least 10 realizations. **C:** Average time t_{fix} to fixation of the C microorganisms versus alternation period T for various carrying capacities K. Data shown if resistance took over in at least 10 realizations. Horizontal solid lines: analytical predictions for very small T, using the self-averaged fitness $\tilde{f}_S$ (see main text). In panels **B** and **C**, markers are averages over $10^2 - 10^3$ simulation realizations, error bars (often smaller than markers) represent 95% confidence intervals, and the oblique black line corresponds to $T/2$. In all panels, colored dashed lines correspond to $T/2 = \tau_S$, while black dashed lines correspond to $T/2 = \tau_V$. Parameter values: $f_S = 1$ without antimicrobial, $f'_S = 0$ with antimicrobial, $f_R = 0.9$, $f_C = 1$, $g_S = g_R = g_C = 0.1$, $\mu_1 = 10^{-5}$ and $\mu_2 = 10^{-3}$. All simulations start with 10 S microorganisms.

2.2 Biocidal antimicrobial

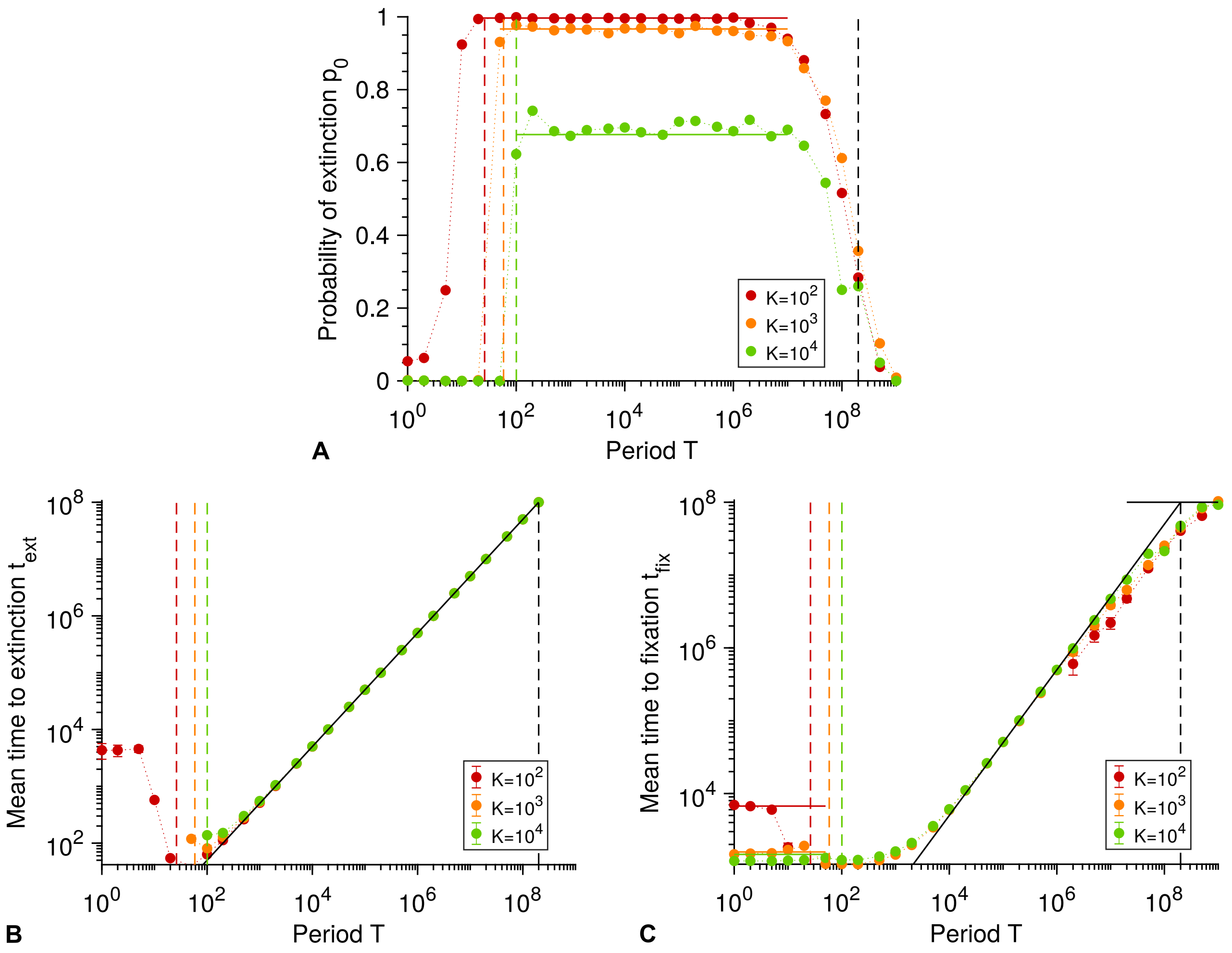

Fig V. Periodic presence of a biocidal antimicrobial above the MIC, including long periods. A: Probability p_0 that the microbial population goes extinct before resistance gets established versus alternation period T, for various carrying capacities K. Markers: simulation results, with probabilities estimated over $10^2 - 10^3$ realizations. Horizontal solid lines: analytical predictions from Eq. 4. **B:** Average time t_{ext} to extinction versus alternation period T for various carrying capacities K. Data shown if extinction occurred in at least 10 realizations. **C:** Average time t_{fix} to fixation of the C microorganisms versus alternation period T for various carrying capacities K. Data shown if resistance took over in at least 10 realizations. Horizontal solid colored lines: analytical predictions for very small T, using the self-averaged death rate $\tilde{g}_S$ (see below). Horizontal solid black line: average spontaneous valley crossing time $\tau_V = (f_S - f_R)/(\mu_1\mu_2 g_S)$ (see main text). In panels **B** and **C**, markers are averages over $10^2 - 10^3$ simulation realizations, error bars (often smaller than markers) represent 95% confidence intervals, and the oblique black line corresponds to $T/2$. In all panels, colored dashed lines correspond to $T/2 = \tau_S$, while black dashed lines correspond to $T/2 = \tau_V$. Parameter values: $f_S = 1$, $f_R = 0.9$, $f_C = 1$, $g_S = 0.1$ without antimicrobial, $g'_S = 1.1$ with antimicrobial, $g_R = g_C = 0.1$, $\mu_1 = 10^{-5}$ and $\mu_2 = 10^{-3}$. All simulations start with 10 S microorganisms.

Here, in the limit of very fast alternations, we expect an effective averaging of death rates, with $\tilde{g}_S = 0.6$ for S microorganisms. Then, an R mutant that will fix in the population appears after an average time $\tilde{t}^a_R = 1/(\tilde{N}\mu_1\tilde{g}_S\tilde{p}_{SR})$ where $\tilde{N}\mu_1\tilde{g}_S$ represents the total mutation rate in the population, with $\tilde{N} = K(1-\tilde{g}_S/f_S)$ the equilibrium population size, and where $\tilde{p}_{SR} = [1 - f_S g_R/(f_R\tilde{g}_S)]/[1 - (f_S g_R/(f_R\tilde{g}_S))^{\tilde{N}}]$ is the probability that a single R mutant fixes in a population of $\tilde{N}$ microorganisms where all other microorganisms are S. Subsequently, C mutants will appear and

fix, thus leading to the full evolution of resistance by the population. The corresponding average total time t_{fix} of resistance evolution [1] agrees well with simulation results for $T/2 \ll \tau_S$ (see Fig. VC).

2.3 Population size dependence of the extinction transition

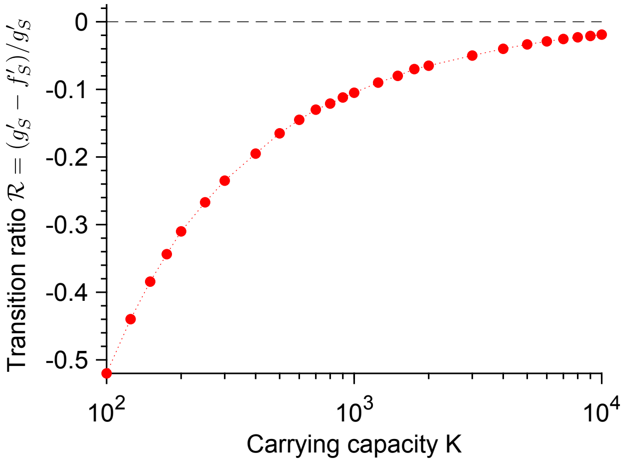

Fig VI. Finite size effect on the extinction transition. Value of the ratio $\mathcal{R} = (g'_S - f'_S)/g'_S$ such that $t^a_R = \tau_S$, plotted versus the carrying capacity K. This value of $\mathcal{R}$ marks the transition between large and small extinction probability p_0 when $T/2 > \tau_S$ (see main text and Fig. 5). Red markers: numerical solutions of the equation $t^a_R = \tau_S$. Black dashed line: expected transition in the large population limit ($\mathcal{R} = 0$, i.e. $f'_S = g'_S$). Parameter values: $\mu_1 = 10^{-5}$, $f_S = 1$, $f_R = 0.9$, $g_S = g_R = 0.1$. Here, results are shown in the biostatic case, and f'_S was varied, keeping $g'_S = 0.1$, but the biocidal case yields the exact same results (see main text).

2.4 Dependence of the extinction time on population size and antimicrobial mode of action

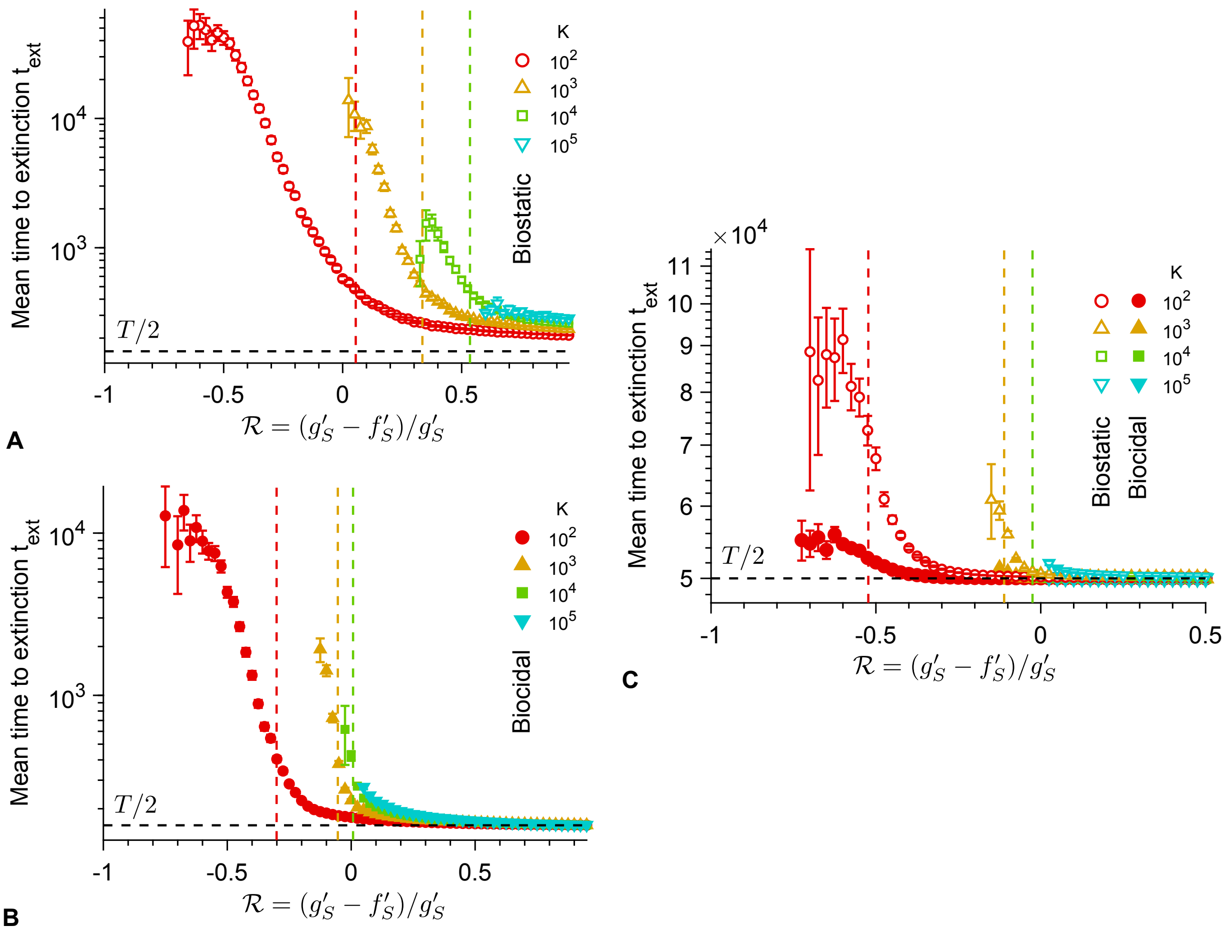

Fig VII. Dependence of the average extinction time on population size and antimicrobial mode of action. Average extinction time t_{ext} versus the ratio $\mathcal{R} = (g'_S - f'_S)/g'_S$ with biostatic or biocidal antimicrobial, for different carrying capacities K, either in the small-period regime, with $T = 10^{2.5}$ (**A** and **B**) or in the large-period regime, with $T = 10^5$ (**C**). Markers: simulation results, calculated over the realizations ending in extinction of the population, if their number is at least 10, among 10^3 realizations total per marker. Error bars: 95% confidence intervals. Vertical dashed lines: predicted extinction thresholds, i.e. values of $\mathcal{R}$ such that $T/2 = \tau_S$ (**A** and **B**) or $t_R^a = \tau_S$ (**C**). Horizontal dashed lines: $t_{ext} = T/2$. Parameter values (same as in Fig. 6): $\mu_1 = 10^{-5}$, $\mu_2 = 10^{-3}$, $f_S = 1$, $f_R = 0.9$, $f_C = 1$, $g_S = g_R = g_C = 0.1$, and $g'_S = 0.1$ (biostatic) or $f'_S = 1$ (biocidal). All simulations start with 10 S microorganisms.

3 Rescue by resistance

3.1 Number of resistant mutants when antimicrobial is added: $p_R^c(i)$

Let $p_R^c(i)$ be the probability that exactly i R microorganisms are present when antimicrobial is added, provided that a lineage of R mutants then exists. It can be calculated in the framework of the Moran model, provided that the population size is stable around $N = K(1 - g_S/f_S)$ before antimicrobial is added, which is correct for $T/2 \gg t_r$, where t_r is the rise time (see section 1.3.1). Specifically, $p_R^c(i)$ can be expressed as a ratio of the sojourn time in state i to the total lifetime of the lineage in the absence of antimicrobial:

$$p_R^c(i) = \frac{\tau_{R,i}^d}{\tau_R^d}\,, \tag{S17}$$

where τ_R^d is the average lifetime without antimicrobial of the lineage of a resistant mutant, assuming that it is destined for extinction, and $\tau_{R,i}^d$ is the average time this lineage spends with exactly i R individuals before going extinct. They satisfy $\tau_R^d = \sum_{i=1}^{N-1} \tau_{R,i}^d$. Note that we consider lineages destined for extinction in the absence of antimicrobial, because we focus on timescales much shorter than the spontaneous valley crossing time. In fact, in this regime, considering unconditional times yields nearly identical values for $p_R^c(i)$.

Employing the master equation $\dot{\mathbf{P}} = \mathbf{RP}$ that describes the time evolution of the number of R mutants within the Moran model [1, 4], where $\mathbf{R}$ is the transition rate matrix, we obtain

$$\tau_{R,i}^d = \frac{\pi_i}{\pi_1} \int_0^\infty P_i(t)dt = -\frac{\pi_i}{\pi_1} (\tilde{\mathbf{R}}^{-1})_{i\,1}\,, \tag{S18}$$

where π_i is the probability that the R mutants go extinct, starting from i R mutants [1, 4], while $\tilde{\mathbf{R}}$ is the reduced transition rate matrix, which is identical to the transition rate matrix $\mathbf{R}$, except that the rows and the columns corresponding to the absorbing states $i = 0$ and $i = N$ are removed [1]. Here, we take $N = K(1 - g_S/f_S)$, which corresponds to the deterministic equilibrium population size. Finally, we obtain

$$p_R^c(i) = \frac{\pi_i (\tilde{\mathbf{R}}^{-1})_{i\,1}}{\sum_{k=1}^{N-1} \pi_k (\tilde{\mathbf{R}}^{-1})_{k\,1}}\,. \tag{S19}$$

3.2 Probability of fast extinction of the resistant mutants: $p_R^e(i)$

Let us consider the beginning of the first phase with antimicrobial, and take as our origin of time $t = 0$ the beginning of the phase with antimicrobial. Here, we consider the general case of an antimicrobial that may modify both the division rate and the death rate of sensitive microorganisms. Provided that some resistant microorganisms are present at $t = 0$, how likely is it that they will undergo a rapid stochastic extinction and not rescue the microbial population and lead to the establishment of resistance? Denoting by $i > 0$ the number of resistant microorganisms at $t = 0$, let us estimate the probability $p_R^e(i)$ that the lineage of R mutants then quickly goes extinct. As explained in the main text, we approximate the reproduction rate of the R microorganisms by

$$f_R(t) = f_R \left(1 - \frac{S(t) + R(t)}{K}\right) \approx f_R \left(1 - \frac{S(t)}{K}\right)\,, \tag{S20}$$

where $S(t)$ and $R(t)$ are the numbers of S and R individuals at time t. This is appropriate because early extinctions of R mutants tend to happen shortly after the addition of antimicrobials, when $S(t) \gg R(t)$. Thus motivated, we further employ the deterministic approximation to describe the decreasing number $S(t)$ of S microorganisms:

$$S(t) = \frac{K(1 - g'_S/f'_S) S_0 e^{(f'_S - g'_S)t}}{K(1 - g'_S/f'_S) + S_0 (e^{(f'_S - g'_S)t} - 1)}\,, \tag{S21}$$

where $S_0 = K(1 - g_S/f_S)$ is the number of sensitive microorganisms when antimicrobial is added. Note that if $f'_S = 0$ and $g'_S = g_S$, i.e. in the perfect biostatic case, we obtain

$$S(t) = K\left(1 - \frac{g_S}{f_S}\right) e^{-g_S t}\,, \tag{S22}$$

for the decay of the number of S microorganisms with antimicrobial. However, we retain a stochastic description for the rare R mutants, and employ the probability generating function

$$\phi_i(z,t) = \sum_{j=0}^{\infty} z^j P(j,t|i,0)\,, \tag{S23}$$

where i is the initial number of R microorganisms. Indeed, noticing that

$$p_R^e(i) = \lim_{t\to\infty} P(0,t|i,0) = \lim_{t\to\infty} \phi_i(0,t) \tag{S24}$$

will enable us to calculate $p_R^e(i)$ [5,6].

The probability $P(j,t|i,0)$ of having j R mutants at time t, starting from i R mutants at time $t=0$, satisfies the master equation

$$\frac{\partial P(j,t|i,0)}{\partial t} = f_R(t)\,(j-1)\,P(j-1,t|i,0) + g_R\,(j+1)\,P(j+1,t|i,0) - (f_R(t)+g_R)\,j\,P(j,t|i,0)\;. \tag{S25}$$

Here, we neglect mutants that appear after the addition of antimicrobial, and we deal with them in the calculation of p_R^a and $p_R^{e'}$. The generating function defined in Eq. S23 satisfies the partial differential equation

$$\frac{\partial \phi_i(z,t)}{\partial t} - (z-1)(f_R(t)z - g_R)\frac{\partial \phi_i(z,t)}{\partial z} = 0\,. \tag{S26}$$

This first-order nonlinear partial differential equation can be solved using the method of characteristics. For this, we rewrite it as:

$$\vec{v}.\vec{\nabla}\phi_i = 0\;, \tag{S27}$$

where $\vec{v} = (1,\ -(z-1)(f_B(t)z - g_B))^t$ and $\vec{\nabla}\phi_i = (\partial\phi_i/\partial t,\ \partial\phi_i/\partial z)^t$. A characteristic curve $\vec{r}(s)$ satisfies $d\vec{r}/ds = \vec{v}(\vec{r}(s))$, which entails

$$\frac{d\phi_i}{ds} = \frac{d\vec{r}}{ds}.\vec{\nabla}\phi_i = \vec{v}.\vec{\nabla}\phi_i = 0\,, \tag{S28}$$

implying that ϕ_i is constant along a characteristic curve. Since $d\phi_i/ds = (\partial\phi_i/\partial t)(dt/ds) + (\partial\phi_i/\partial z)(dz/ds)$, we obtain the following system of ordinary differential equations along a characteristic curve:

$$\begin{cases} \frac{dt}{ds} = 1\,, \\ \frac{dz}{ds} = -(z-1)(f_R(t)z - g_R)\,. \end{cases} \tag{S29}$$

We choose to integrate it as

$$\begin{cases} t = s\,, \\ \frac{dz}{dt} = -(z-1)(f_R(t)z - g_R)\,. \end{cases} \tag{S30}$$

The second ordinary differential equation can be solved by introducing $y = 1/(z-1)$, which yields

$$\frac{e^{\rho(t)}}{z-1} - \int_0^t f_R(u)e^{\rho(u)}du = \frac{1}{z_0-1}\,, \tag{S31}$$

with

$$\rho(t) = \int_0^t (g_R - f_R(u))\,du\,, \tag{S32}$$

where we have employed Eqs. S20 and S22. Eq. S31 is the equation of the characteristic line going through the point $(0,\ z_0)$. Because ϕ_i is constant along this line (see Eq. S28), we have $\phi_i(z,t) = \phi_i(z_0,0) = z_0^i$ along this line, where we have used Eq. S23. Furthermore, for any (z,t) we can find the appropriate z_0 using Eq. S31. This yields the following expression for the generating function:

$$\phi_i(z,t) = \left[1 + \left(\frac{e^{\rho(t)}}{z-1} - \int_0^t f_R(u)e^{\rho(u)}du\right)^{-1}\right]^i\,, \tag{S33}$$

where $\rho(t)$ is given by Eq. S32 and $f_R(t)$ by Eq. S20.

We can now express the probability $p_R^e(i)$ from Eqs. S24 and S33:

$$p_R^e(i) = \lim_{t\to\infty}\left[\frac{g_R\int_0^t e^{\rho(u)}du}{1 + g_R\int_0^t e^{\rho(u)}du}\right]^i\;. \tag{S34}$$

3.3 Predicting the extinction probability p_0

Here, we test the analytical predictions for each term involved in the extinction probability p_0 of the population above the MIC, both in the perfect biostatic case (see Eq. 1) and in the biocidal case (see Eq. 4), by comparing them to numerical simulation results. To estimate the probability p_R that at least one R mutant is present when antimicrobial is added, and to study the number of R mutants that are then present (Fig. VIIIA-B), simulations are run starting from $j_0 = 10$ S microorganisms (and no R) as in the rest of our work. We let the population evolve until a specific time, in practice $t = 500$, when population size is well-equilibrated around the deterministic stationary value $K(1 - g_S/f_S)$ without antimicrobial, and we analyze population composition at this time. To estimate the probability p_R^e of rapid extinction of the R lineage (Figs. VIIIC and XA), we start from a population with i R microorganisms and $K(1 - g_S/f_S) - i$ sensitive microorganisms, and we let it evolve with antimicrobial until extinction of the S microorganisms. All these simulations are run with 2 types of microorganisms, S and R (no compensation). In Figs. VIIIC and XA, we note that p_R^e does not seem to depend on K. In fact, our analytical estimate for p_R^e is fully independent of K because it only involves the ratio $S(t)/K$ (see Eqs. S34, S32 and S20), whose deterministic dynamics is independent of K (see Eq. S9 with $N(t) \leftarrow S(t)$).

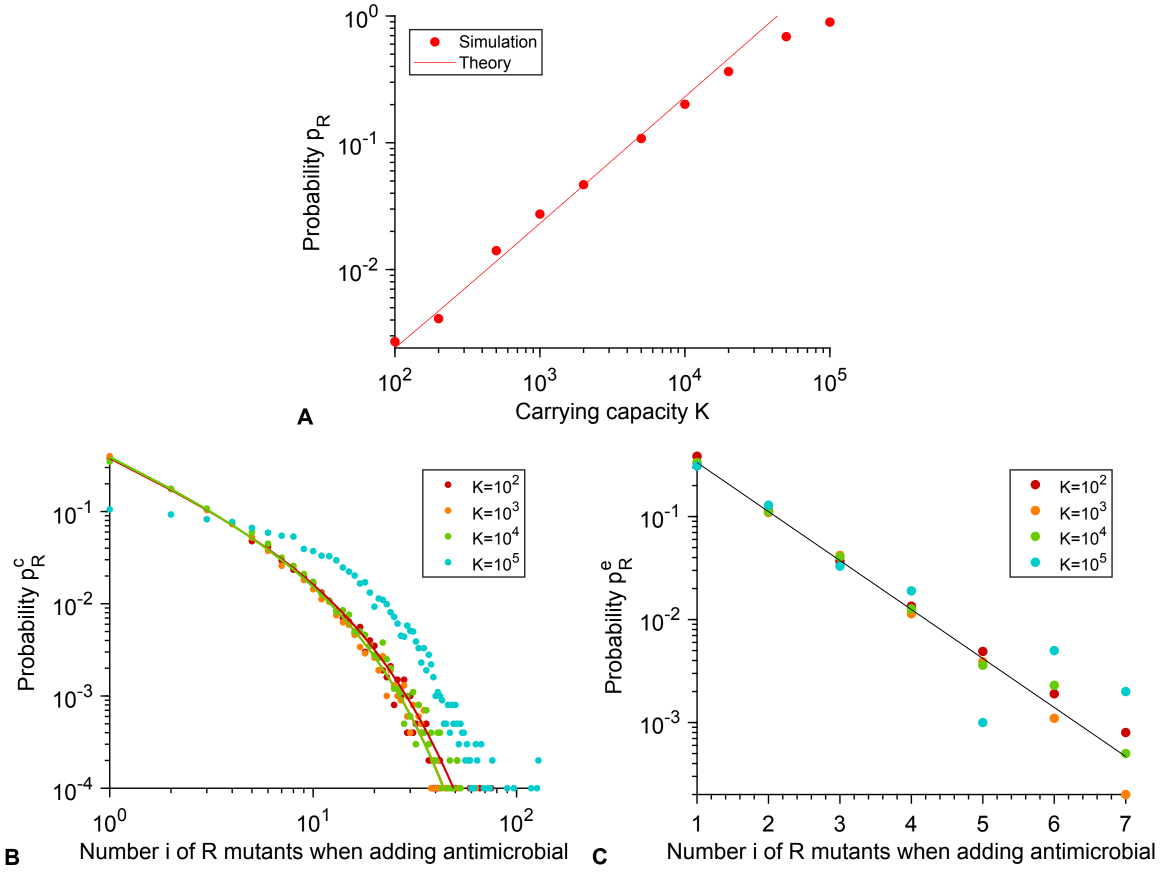

Fig VIII. Perfect biostatic antimicrobial: test of analytical predictions for each term involved in p_0 (Eq. 1). A: Probability p_R that at least one R mutant is present when antimicrobial is added, plotted versus carrying capacity K. Markers: simulation results, with probabilities estimated over 10^4 realizations. Red solid line: analytical prediction, $p_R = t_R^{app}/\tau_R^d = N\mu_1 g_S \tau_R^d$ (see main text). **B:** Probability p_R^c that exactly i R microorganisms are present when antimicrobial is added, provided that at least one R mutant is present, plotted versus the number i of R mutants, for various carrying capacities K. Markers: simulation results, estimated over 10^4 realizations. Solid lines: analytical prediction in Eq. S19. Analytical prediction lines for $K = 10^4$ and $K = 10^5$ are confounded; note that the prediction holds in the weak mutation regime $K\mu_1 \ll 1$, and thus fails for $K = 10^5$ here. **C:** Probability p_R^e of rapid extinction of the R lineage, plotted versus the number i of R mutants present when adding antimicrobial, for various different carrying capacities K. Markers: simulation results, with probabilities estimated over 10^4 realizations. Black solid line: analytical prediction from Eq. 2 (see main text). Parameter values: $f_S = 1$ without antimicrobial, $f'_S = 0$ with antimicrobial, $f_R = 0.9$, $g_S = g_R = 0.1$ and $\mu_1 = 10^{-5}$ (**A-B**) or $\mu_1 = 0$ (**C**).

The probability p_R^a that resistance appears in the presence of antimicrobial involves the number of divisions N_{div} and the mean time to extinction τ_S of a population of S microorganisms in the presence of antimicrobial (see main text). To estimate these two intermediate quantities, simulations only involving S microorganisms in the presence of antimicrobial, starting from $K(1 - g_S/f_S)$ sensitive microorganisms, are performed (Fig. IXA-B). For p_R^a itself (Fig. XB), simulations with S and R microbes (no compensation), also starting from $K(1 - g_S/f_S)$ sensitive microorganisms in the presence of antimicrobial, are performed. The time of appearance of R mutants (Fig. IXC-D) and the number of different lineages that appear during the decay of this population (Fig. XC) are also studied.

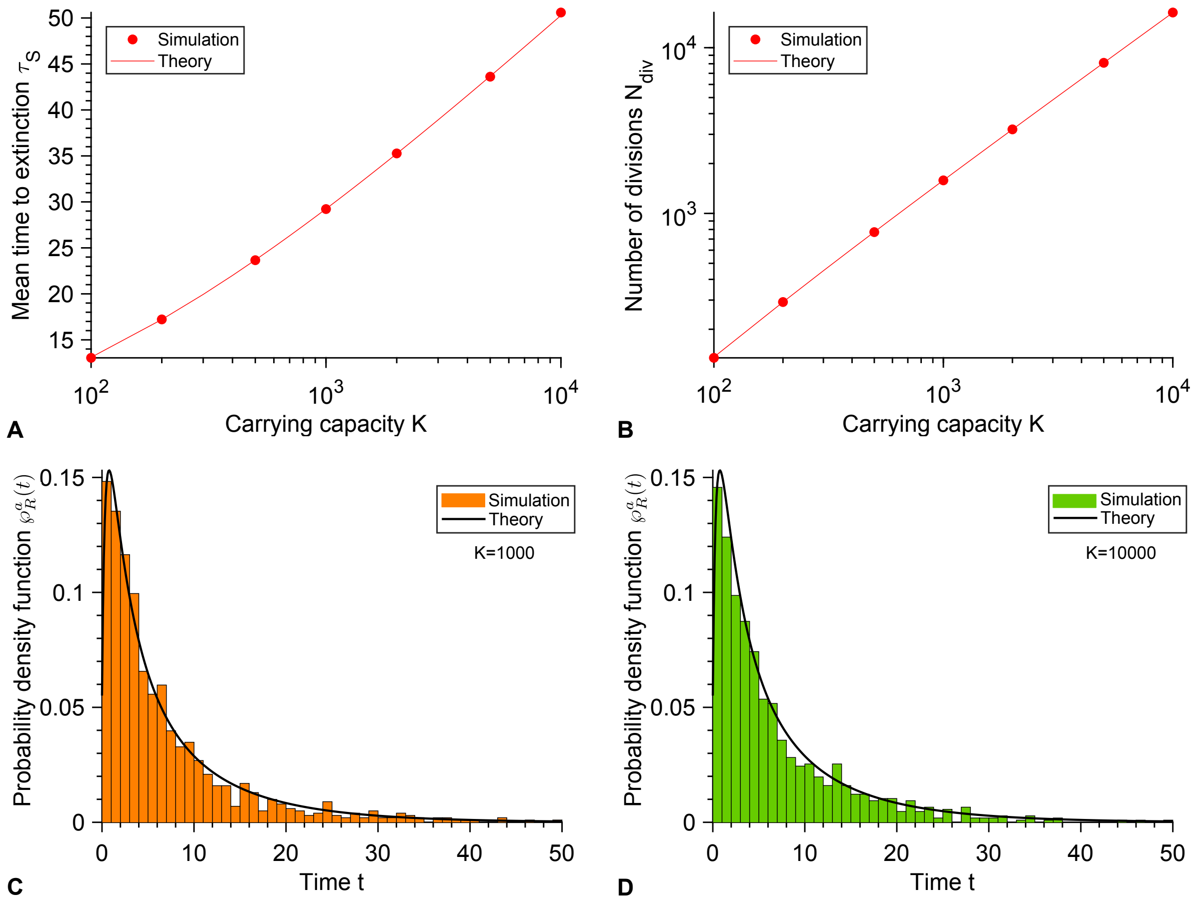

Fig IX. Biocidal antimicrobial: test of analytical predictions for intermediate quantities involved in the calculation of p_0 (see Eq. 4). A: Average time τ_S to extinction of a population of S microorganisms in the presence of antimicrobial, plotted versus the carrying capacity K. Markers: simulation results, with probabilities estimated over 10^4 realizations. Red solid line: analytical prediction from Eq. S6, with $j_0 = K(1 - g_S/f_S)$. **B:** Number N_{div} of individual division events that occur between the addition of antimicrobial and the extinction of the population of S microorganisms, plotted versus carrying capacity K. Red markers: simulation results, with probabilities estimated over 10^4 realizations. Red solid line: analytical prediction from Eq. 5. **C and D:** Probability density function $\wp_R^a(t)$ of the time t of appearance of an R mutant, under the assumption that exactly one R mutant appears between the addition of antimicrobial and the extinction of the population of S microorganisms, for $K = 10^3$ (**C**) and $K = 10^4$ (**D**). Histograms: simulation results, with 10^3 realizations. Black solid lines: analytical prediction from Eq. 9. Parameter values: $f_S = 1$, $g_S = 0.1$ without antimicrobial, $g'_S = 1.1$ with antimicrobial, and in panels **C** and **D**, $f_R = 0.9$, $g_R = 0.1$ and $\mu_1 = 10^{-5}$.

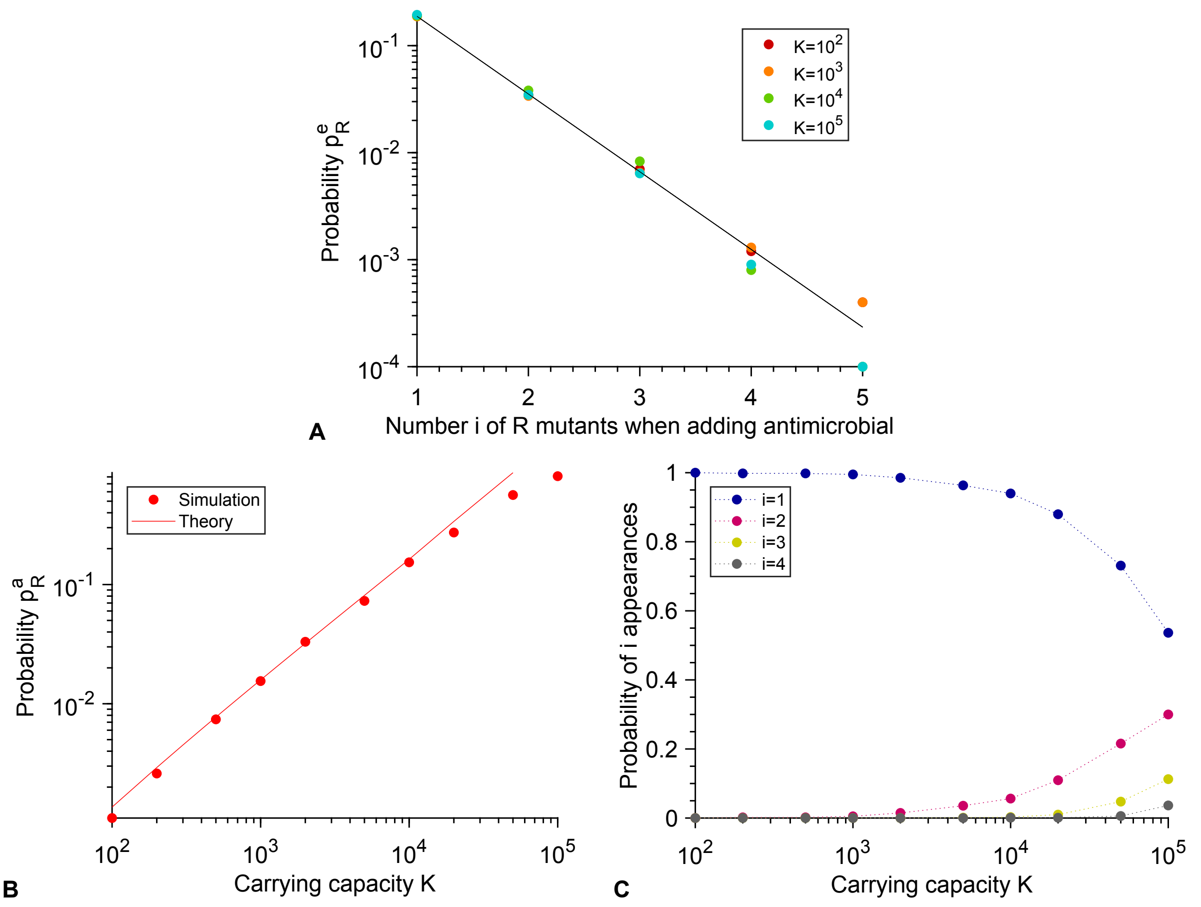

Fig X. Biocidal antimicrobial: test of analytical predictions for each term involved in p_0 (see Eq. 4). Note that p_R and p_R^c are the same as in Fig. VIIIA-B. **A:** Probability p_R^e of rapid extinction of the R lineage, plotted versus the number i of R mutants present when adding antimicrobial, for various different carrying capacities K. Markers: simulation results, with probabilities estimated over 10^4 realizations. Black solid line: analytical prediction from Eq. S34. **B:** Probability p_R^a that resistance appears in the presence of antimicrobial, plotted versus the carrying capacity K. Red markers: simulation results, with probabilities estimated over 10^4 realizations. Red solid line: analytical prediction, $p_R^a = N_{div}\mu_1$ with N_{div} in Eq. 5. **C:** Probability that i distinct lineages of R mutants appear in the presence of antimicrobial, provided that at least one appears, plotted versus the carrying capacity K. Markers: simulation results, with probabilities estimated over 10^3 realizations. Parameter values: $f_S = 1$, $f_R = 0.9$, $g_S = 0.1$ without antimicrobial, $g_S' = 1.1$ with antimicrobial, $g_R = 0.1$ and $\mu_1 = 0$ (panel **A**) or $\mu_1 = 10^{-5}$ (panels **B** and **C**).

3.4 A perfect biostatic antimicrobial yields a larger p_0 than a perfect biocidal antimicrobial

For a perfect biostatic antimicrobial, the extinction probability p_0 upon the first addition of drug is given by Eq. 1:

$$p_0 = 1 - p_R \sum_{i=1}^{N-1} p_R^c(i)(1 - p_R^e(i)) \,, \tag{S35}$$

while for a biocidal antimicrobial, the extinction probability $\tilde{p}_0$ upon the first addition of drug is given by Eq. 4:

$$\tilde{p}_0 = \left[1 - p_R \sum_{i=1}^{N-1} p_R^c(i)(1 - \tilde{p}_R^e(i))\right] \left[1 - p_R^a(1 - p_R^{e'})\right] < 1 - p_R \sum_{i=1}^{N-1} p_R^c(i)(1 - \tilde{p}_R^e(i)) \,. \tag{S36}$$

In Eq. S36 we have employed tilde symbols to denote the quantities that differ compared to Eq. S35. Recall that p_R and $p_R^c(i)$ are the same in both cases. Indeed, these quantities characterize the state of the population when the antimicrobial is added, and thus do not depend on the type of treatment subsequently added.

The perfect biocidal antimicrobial corresponds to $g'_S \to \infty$. Let us prove that $\lim_{g'_S \to \infty} \tilde{p}_0 < p_0$. From Eqs. S35 and S36 it is apparent that it suffices to prove that $\lim_{g'_S \to \infty} \tilde{p}_R^e(i) < p_R^e(i)$ for all i. The expression of both $p_R^e(i)$ and $\tilde{p}_R^e(i)$ is given in Eq. S34, but it involves the decaying number $S(t)$ of S microorganisms once antimicrobial is added, which is different in these two cases, and is given respectively by Eq. S21 with $f'_S = f_S$ in the biocidal case and by Eq. S22 in the perfect biostatic case.

Taking the limit $g'_S \to \infty$ in Eq. S34 yields $\lim_{g'_S \to \infty} \tilde{p}_R^e(i) = (g_R/f_R)^i$, which corresponds to the extinction probability of a population that starts from i R microorganisms, in the absence of any other microorganisms [7]. But for a perfect biostatic antimicrobial,

$$\rho(t) = \int_0^t \left[g_R - f_R\left(1 - \frac{S(u)}{K}\right)\right] du > \int_0^t [g_R - f_R]\, du = (g_R - f_R)t \,, \tag{S37}$$

which, using Eq. S34, entails that $p_R^e(i) > (g_R/f_R)^i$, i.e. $\lim_{g'_S \to \infty} \tilde{p}_R^e(i) < p_R^e(i)$ for all i. Therefore, we have shown that $\lim_{g'_S \to \infty} \tilde{p}_0 < p_0$: the extinction probability p_0 is larger for a perfect biostatic antimicrobial than for a perfect biocidal antimicrobial.

Importantly, our proof does not rely on the appearance of resistant microorganisms while antimicrobial is present, which cannot happen with a perfect biostatic antimicrobial, and whose probability tends to zero when $g'_S \to \infty$ with a biocidal antimicrobial. What makes the perfect biostatic antimicrobial more efficient than the perfect biocidal one is that S microorganisms survive for a longer time, thereby reducing the division rate of R microorganisms due to the logistic term, and favoring their extinction. Such a competition effect is realistic if S microorganisms still take up resources (e.g. nutrients) even while they are not dividing.

4 Fixation probability of a mutant in a population of constant size

In the main text, in our discussion of sub-MIC concentrations of antimicrobials, we employed the fixation probability p_{SR} of an R mutant in a population of S individuals with fixed size N:

$$p_{SR} = \frac{1 - f_S g_R/(f_R g_S)}{1 - [f_S g_R/(f_R g_S)]^N} \ . \tag{S38}$$

Here, we briefly justify this formula.

Consider a birth-death process in which, at each discrete time step, one individual is chosen with a probability proportional to its fitness to reproduce and another one is chosen with a probability proportional to its death rate to die. Note that at each time step, the total number of individuals in the population stays constant. This model is a variant of the Moran model with selection both on division and on death. Let i be the number of R microorganisms and $N - i$ the number of S microorganisms. At a given time step, the probability T_i^+ that the number of R individuals increases from i to $i + 1$ satisfies:

$$T_i^+ = \frac{f_R i}{f_R i + f_S(N-i)} \frac{g_S(N-i)}{g_R i + g_S(N-i)} \ , \tag{S39}$$

and similarly, the probability T_i^- that i decreases by 1 is given by:

$$T_i^- = \frac{f_S(N-i)}{f_R i + f_S(N-i)} \frac{g_R i}{g_R i + g_S(N-i)} \ . \tag{S40}$$

The probability p_{SR} that the R genotype fixes in the population, starting from 1 R microorganism, then satisfies [8]:

$$p_{SR} = \frac{1}{1 + \sum_{k=1}^{N-1} \prod_{j=1}^{k} \gamma_j} \ , \tag{S41}$$

where

$$\gamma_i = \frac{T_i^-}{T_i^+} = \frac{f_S g_R}{f_R g_S} \ . \tag{S42}$$

We thus obtain the result announced in Eq. S38.

5 Detailed simulation methods

In this work, the evolution of microbial populations are simulated using a Gillespie algorithm [9, 10]. Let us denote by j_S, j_R and j_C the respective numbers of S, R and C individuals. The elementary events that can happen are division with or without mutation and death of an individual microbe of either type:

- $S \xrightarrow{k_S^+} 2S$: Reproduction without mutation of a sensitive microbe with rate $k_S^+ = f_S^e(1-(j_S+j_R+j_C)/K)(1-\mu_1)$, with $f_S^e = f_S$ if no antimicrobial is present in the environment or $f_S^e = f'_S$ if antimicrobial is present in the environment.
- $S \xrightarrow{k_{SR}} S+R$: Reproduction with mutation of a sensitive microbe with rate $k_{SR} = f_S^e(1-(j_S+j_R+j_C)/K)\mu_1$.
- $S \xrightarrow{k_S^-} \emptyset$: Death of a sensitive microbe with rate $k_S^- = g_S^e$, with $g_S^e = g_S$ if no antimicrobial is present in the environment or $g_S^e = g'_S$ if antimicrobial is present in the environment.
- $R \xrightarrow{k_R^+} 2R$: Reproduction without mutation of a resistant microbe with rate $k_R^+ = f_R(1-(j_S+j_R+j_C)/K)(1-\mu_2)$.
- $R \xrightarrow{k_{RC}} R+C$: Reproduction with mutation of a resistant microbe with rate $k_{RC} = f_R(1-(j_S+j_R+j_C)/K)\mu_2$.
- $R \xrightarrow{k_R^-} \emptyset$: Death of a resistant microbe with rate $k_R^- = g_R$.
- $C \xrightarrow{k_C^+} 2C$: Reproduction of a resistant-compensated microbe with rate $k_C^+ = f_C(1-(j_S+j_R+j_C)/K)$.
- $C \xrightarrow{k_C^-} \emptyset$: Death of a resistant-compensated microbe with rate $k_C^- = g_C$.

The total rate of events is given by $k_{tot} = (k_S^+ + k_{SR} + k_S^-)j_S + (k_R^+ + k_{RC} + k_R^-)j_R + (k_C^+ + k_C^-)j_C$.

Simulation steps are as follows:

1. Initialization: The microbial population starts from $j_S = 10$ sensitive microorganisms, $j_R = 0$ resistant mutant and $j_C = 0$ resistant-compensated mutant at time $t = 0$ without antimicrobial. The next time when the environment changes is stored in the variable t_{switch}, which is initialized at $t_{switch} = T/2$, the first time when antimicrobial is added.
2. The time increment Δt is sampled randomly from an exponential distribution with mean $1/k_{tot}$, and the next event that may occur is chosen randomly, proportionally to its probability k/k_{tot}, where k is its rate. For instance, division of a sensitive microorganism without mutation is chosen with probability $k_S^+ j_S/k_{tot}$.
3. If $t + \Delta t < t_{switch}$, time is increased to $t + \Delta t$ and the event chosen at Step 2 is executed.
4. If $t + \Delta t \geq t_{switch}$, the event chosen at Step 2 is not executed, because an environment change has to occur before. The environment change is performed: time is incremented to $t = t_{switch}$, and the fitness and death rate of the sensitive microbes are switched from f_S to f'_S and from g_S to g'_S or vice-versa. In addition, t_{switch} is incremented to $t_{switch} + T/2$, and thus stores the next time when the environment changes.
5. We go back to Step 2 and iterate until the total number of microbes is zero ($j_S + j_R + j_C = 0$) or there are only resistant-compensated mutants ($j_S = 0$, $j_R = 0$ and $j_C \neq 0$).

Note that Step 4 introduces an artificial discretization of time, because environment changes occur at fixed times and not with a fixed rate. However, because the total event rate is large unless the population size is very small, the "jump" in time induced by Step 4 is usually extremely small, and the discarded events constitute a tiny minority of events. The resulting error is thus expected to be negligible. The very good agreement between our simulation results and our analytical predictions, in particular for short periods, corroborates this point.

References

1. Marrec L, Bitbol AF. Quantifying the impact of a periodic presence of antimicrobial on resistance evolution in a homogeneous microbial population of fixed size. J Theor Biol. 2018;457:190–198.

2. Sekimoto K. Stochastic Energetics. Springer-Verlag; 2010.

3. Kendall DG. On the Generalized “Birth-and-Death” Process. Ann Math Statist. 1948;19(1):1–15.

4. Ewens WJ. Mathematical Population Genetics. Springer-Verlag; 1979.

5. Alexander HK, Bonhoeffer S. Pre-existence and emergence of drug resistance in a generalized model of intra-host viral dynamics. Epidemics. 2012;4:187–202.

6. Parzen E. Stochastic processes. SIAM, Philadelphia; 1999.

7. Coates J, Park BR, Le D, Simsek E, Chaudhry W, Kim M. Antibiotic-induced population fluctuations and stochastic clearance of bacteria. Elife. 2018;7.

8. Traulsen A, Hauert C. Stochastic evolutionary game dynamics. In: Schuster HG, editor. Reviews of Nonlinear Dynamics and Complexity. vol. II. Wiley-VCH; 2009.

9. Gillespie DT. A general method for numerically simulating the stochastic time evolution of coupled chemical reactions. J Comput Phys. 1976;22:403–434.

10. Gillespie DT. Exact stochastic simulation of coupled chemical reactions. J Phys Chem. 1977;81:2340–2361.
